# Mitochondrion-targeted antioxidant SkQ1 prevents rapid animal death caused by highly diverse shocks

**DOI:** 10.1038/s41598-023-31281-9

**Published:** 2023-03-15

**Authors:** V. P. Skulachev, M. Yu. Vyssokikh, B. V. Chernyak, O. A. Averina, A. A. Andreev-Andrievskiy, R. A. Zinovkin, K. G. Lyamzaev, M. V. Marey, M. V. Egorov, O. J. Frolova, D. B. Zorov, M. V. Skulachev, V. A. Sadovnichii

**Affiliations:** 1 Belozersky Institute of Physico-Chemical Biology, Lomonosov Moscow State University, Moscow, Russia 119991; 2 Institute of Mitoengineering, Lomonosov Moscow State University, Moscow, Russia 119991; 3 Faculty of Biology, Lomonosov Moscow State University, Moscow, Russia 119991; 4 Research Center for Obstetrics, Gynecology and Perinatology, Moscow, Russia 117198; 5 Faculty of Mechanics and Mathematics, Lomonosov Moscow State University, Moscow, Russia 119991

**Keywords:** Ageing, Diseases, Immunology, Cytokines, Inflammation, Drug discovery

## Abstract

The response to stress involves the activation of pathways leading either to protection from the stress origin, eventually resulting in development of stress resistance, or activation of the rapid death of the organism. Here we hypothesize that mitochondrial reactive oxygen species (mtROS) play a key role in stress-induced programmed death of the organism, which we called “phenoptosis” in 1997. We demonstrate that the synthetic mitochondria-targeted antioxidant SkQ1 (which specifically abolishes mtROS) prevents rapid death of mice caused by four mechanistically very different shocks: (a) bacterial lipopolysaccharide (LPS) shock, (b) shock in response to intravenous mitochondrial injection, (c) cold shock, and (d) toxic shock caused by the penetrating cation C_12_TPP. Importantly, under all these stresses mortality was associated with a strong elevation of the levels of pro-inflammatory cytokines and administration of SkQ1 was able to switch off the cytokine storms. Since the main effect of SkQ1 is the neutralization of mtROS, this study provides evidence for the role of mtROS in the activation of innate immune responses mediating stress-induced death of the organism. We propose that SkQ1 may be used clinically to support patients in critical conditions, such as septic shock, extensive trauma, cooling, and severe infection by bacteria or viruses.

## Introduction

The main functions of mitochondria are the production of ATP and various important metabolites, as well as reactive oxygen species (ROS). These functions make mitochondria the central node that regulates cellular metabolism, cellular signaling, and cell death^1–4^. In normal physiology, mitochondria function as important sensors of cellular stresses such as nutrient deprivation, oxidative stress, and mitochondrial unfolded protein response, promoting environmental adaptation. If adaptation mechanisms cannot fully compensate for stressors, mitochondria are involved in the development of pathologies, thus becoming important targets for therapeutic interventions^5,6^. Mitochondrial dysfunction contributes to a variety of pathologies including neurodegeneration, ischemic strokes, immune disorders, etc., as well as aging. Thus, drugs designed to affect mitochondrial function offer a promising approach towards the treatment of multiple diseases^7^.

**Figure 1:**
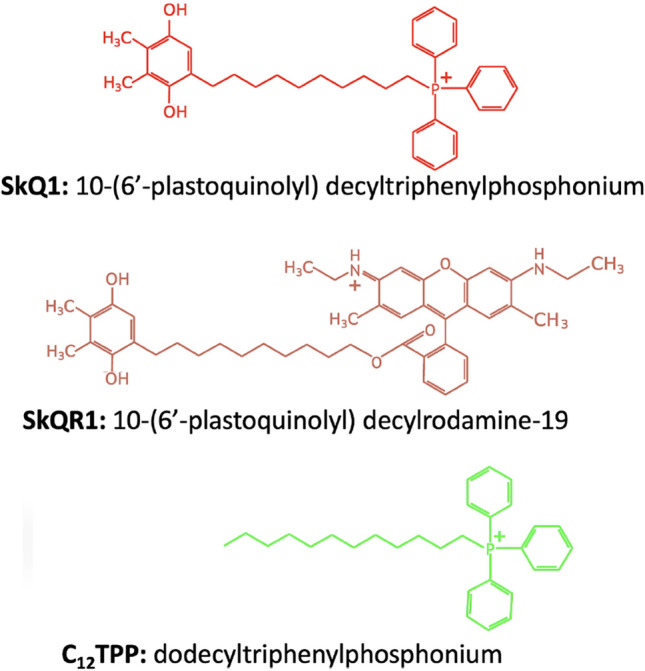
Chemical structure of mitochondria-targeted compounds.

Since excessive production of mitochondrial ROS (mtROS) appears to associate with various pathologies, we and other researchers have developed antioxidant molecules specifically targeted to mitochondria^8,9^. We have developed mitochondria-targeted antioxidants of the SkQ family based on plastoquinol, a very effective antioxidant originated from chloroplasts and cyanobacteria, which can be reduced in mitochondria after oxidation by mtROS. To target plastoquinol into mitochondria, it is conjugated to a cationic residue capable of permeating the mitochondrial membrane. In the case of SkQ1, the phosphorus cation bound to three phenyl rings (triphenylphosphonium, TPP^+^) is conjugated to plastoquinol via a decyl linker. The binding of the cation to phenyls ensures the ability of SkQ1 to penetrate membranes. A positive electrical charge leads to a thousand-fold accumulation of SkQ1 in the inner layer of the inner mitochondrial membrane. In some cases, we used SkQR1 where TPP^+^ was replaced by the nitrogen cation of fluorescent rhodamine 19. Dodecyltriphenylphosphonium (C_12_TPP) lacking plastoquinol serves as a membrane permeant cation without antioxidant function (Fig. 1).

Since 2008, groups involved in our research program and independent laboratories have demonstrated very effective protective effects of SkQ1 and SkQR1 against various pathologies in animal models^10–13^. For example, one of our program laboratories (D.B.Z) demonstrated that SkQ1 and SkQR1 can prevent rapid death in the kidney ischemia/reperfusion model^10,14^, ROS levels increase dramatically in kidney tissue during ischemia, and mitochondria-targeted antioxidants prevented this effect. Based on these findings, here, we hypothesize that mtROS-dependent acute kidney injury can be a common mechanism of programmed death of the organism exposed to many types of crises. To test this idea, we analyzed the protective effect of SkQ1 in four different models of lethal shocks, presumably unrelated to each other.

The first shock was induced by lipopolysaccharide (LPS) of the bacterial cell wall, which simulates septic shock leading to the rapid death. LPS is the most important alarm molecule sensed by the host's innate immune system when invaded by Gram-negative bacterial pathogens. LPS localized in the sites of infection in limited quantities, initiates antimicrobial defense mechanisms, which could lead to the elimination of pathogens. However, generalized infection associated with the release of LPS into the bloodstream initiates a massive production of inflammatory mediators (“cytokine storm”), potentially lethal due to the endothelial damage, refractory shock, and multiple organ failure^15,16^.

The second model of the lethal shock is based on injection of isolated mitochondria into the blood. Mitochondria in the circulation are perceived by the body as a signal of severe tissue damage, and the response resembles septic shock^17^. This response depends on a large family of receptors recognizing several mitochondrial components as damage-associated molecular patterns (DAMPs). Some (but not all) of these receptors also recognize pathogen-associated molecular patterns (PAMPs) found in bacteria and viruses, so mitochondrial shock may differ in some details from bacterial sepsis^17–23^.

A third lethal shock was induced by placing mice for 1 h at − 20 °C. Cold shock is associated with the expression of cold shock proteins, a family of RNA/DNA-binding proteins with different functions^24^. Their role in lethal cold shock and the causes of delayed mortality after acute hypothermia are poorly understood. In humans, lethal cold shock (usually caused by sudden immersion in cold water) is accompanied by severe vasoconstriction, tachyarrhythmia, and heart attack^25^. In addition, simultaneous sympathetic and parasympathetic activation (“autonomous conflict”) may be responsible for some deaths from cold shock^26^.

Finally, we studied lethal toxic shock caused by C_12_TPP. This compound has a high lipophilicity, which predicts its strong hepatotoxicity^27^. Acute liver failure is believed to be the leading cause of death due to C_12_TPP.

In the present study, we have demonstrated that SkQ1 effectively prevents the death of mice caused by all four types of stress. These results are discussed within the framework of the concept of programmed death of the organism (phenoptosis), introduced by one of us (VPS) in 1997^28^ and has since received strong experimental support (see “Discussion” section).

## Results

### SkQ1 prevents lethal shock caused by lipopolysaccharide

**Figure 2:**
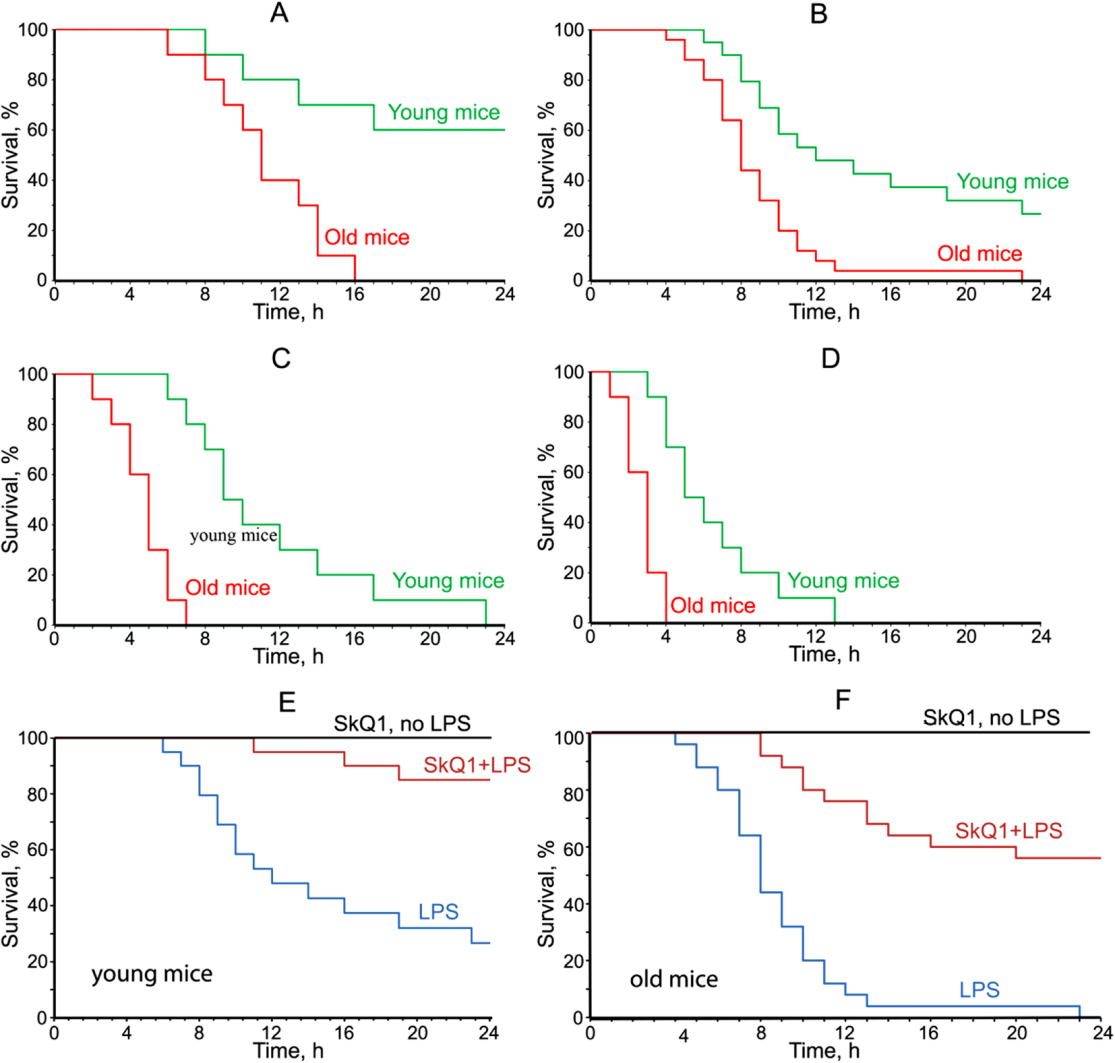
SkQ1 prevents lethal toxicity of bacterial lipopolysaccharide (LPS). LPS at doses10 mg/kg (**A**), 20 mg/kg (**B,E,F**), 30 mg/kg (**C**) or 40 mg/kg, (**D**) was injected intravenously. SkQ1 (1.5 mmol/kg/day) was administered intraperitoneally for 5 days. Here and below Young (3 months) and old (28 months) C57Bl6 mice were used. Kaplan–Meier survival curves are shown. The number of mice in each group treated with LPS was 25, and in groups without LPS, 10.

As shown in Fig. 2A–D, young (3 months old) mice were much more resistant to mortality under LPS than old (28 months old) mice. The results of this experiment were similar to those obtained by Tateda et al.^29^ when the LPS mortality of mice of different ages was studied. Five days of SkQ1 pretreatment strongly prevented subsequent LPS-induced mortality in young animals (Fig. 2F). In old animals (Fig. 2E), the protective effect of SkQ1was also significant. These results were consistent with earlier findings by Zorov et al.^30^ that SkQR1 partially prevented LPS-induced death in 7-day-old newborn rats.

**Figure 3:**
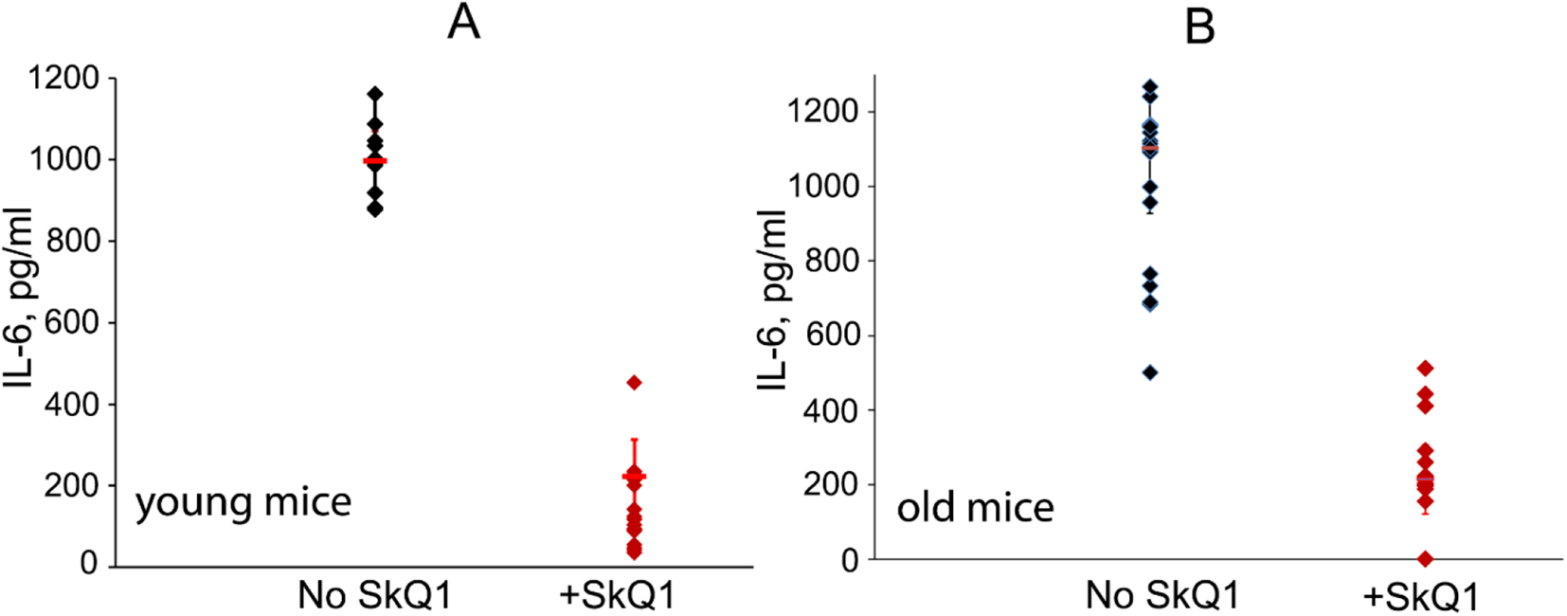
SkQ1 prevents LPS-induced increase in the level of proinflammatory cytokine IL-6. Young (**A**) or old (**B**) mice were injected with LPS (20 mg/kg) and with by SkQ1 (1.5 μmol/kg) as in Fig. 2. The number of mice in each group treated with SkQ1 was 25, and in groups without LPS, 15.

Figure 3 shows that LPS increased the blood level of one of the most important pro-inflammatory cytokines, interleukin IL-6. This effect was prevented by SkQ1 in both young (A) and old (B) mice. These data suggest that the protective effect of SkQ1 was associated with the prevention of the so-called “cytokine storm” when the level of various pro-inflammatory cytokines in the blood rises sharply.

### SkQ1 protects mice from death caused by intravenous injection of mitochondria

**Figure 4:**
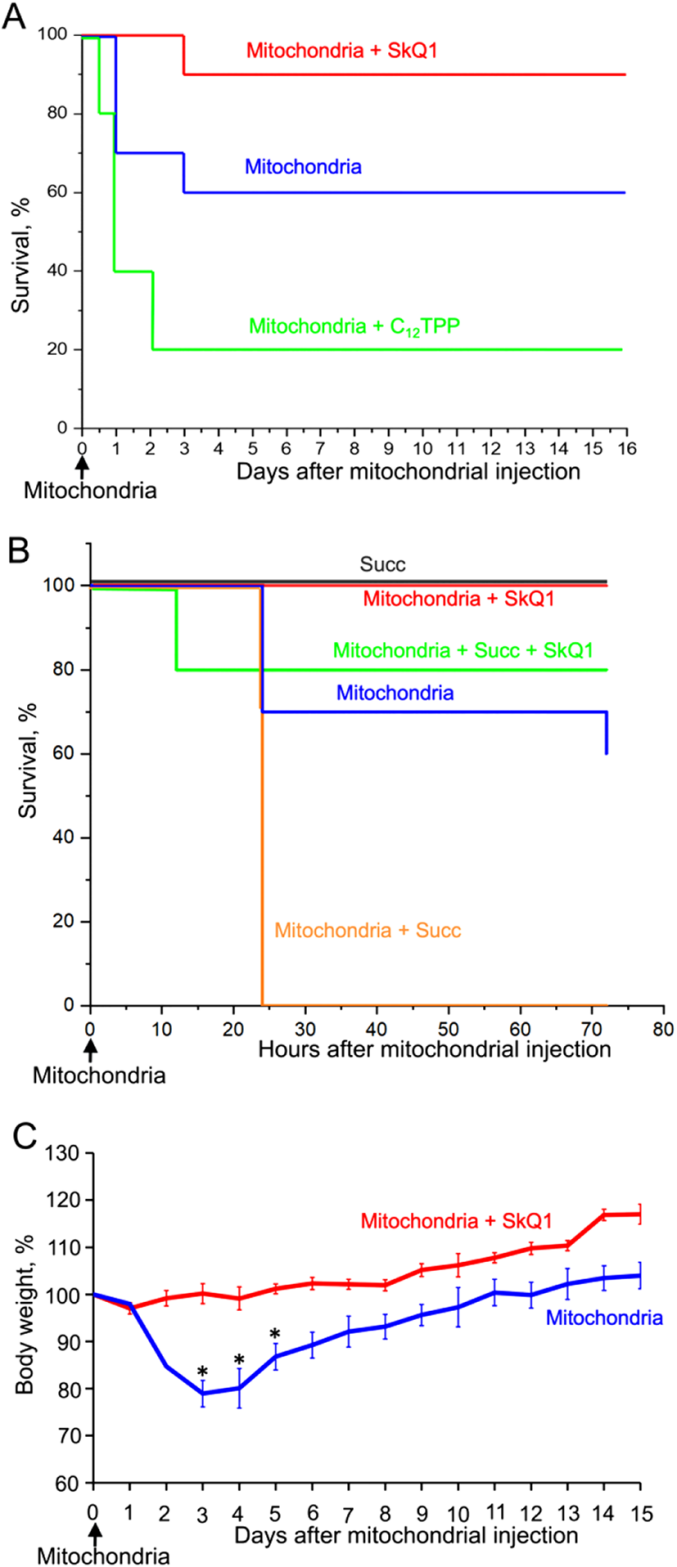
SkQ1 prevents the rapid death of mice after injection of mitochondria (10 mg protein/kg body weight) into the tail vein. (**A–C**) SkQ1 or C_12_TPP (1.5 μmol/kg) was administered intraperitoneally daily for 5 days before and after mitochondrial injection. (**B**) 5 mM succinate (Succ) was added to mitochondria prior to injection. (**C**) Dynamics of mouse body weight after injection of mitochondria. Standard deviations are shown; *p < 0.05. The number of mice in each group treated with SkQ1 was 6, and in groups without SkQ1, 10.

Figure 4A shows the results of an experiment with injection of mouse liver mitochondria. Injection of mitochondria did not decrease survival during the first half-day. However, only 60% of the animals survived the next 2.5 days. A cohort of mice that received the antioxidant SkQ1 daily for five days before mitochondrial injection and then five days after injection demonstrated high resistance to the shock (90% survival). To elucidate the mechanism of SkQ1 action, we compared its effects with effects of C_12_TPP, a SkQ1 analog without antioxidant moiety (plastoquinol). Unlike SkQ1, C_12_TPP even exacerbated the effects of mitochondrial injection, since 20% of mice died in the first half-day. During the next 2 days, the mortality had grown to 80%. Without mitochondrial injection, the same low dose of C_12_TPP proved to have no effect on survival (not shown). Figure 4C and Table S1 show the dynamics of the body weights of mice after intravenous injection of mitochondria. A strong reduction in weight was observed on days 1–3 after mitochondrial injection. Then, the body weight of the surviving animals started to increase. In the presence of SkQ1, we did not observe the weight loss (Fig. 4C).

**Figure 5:**
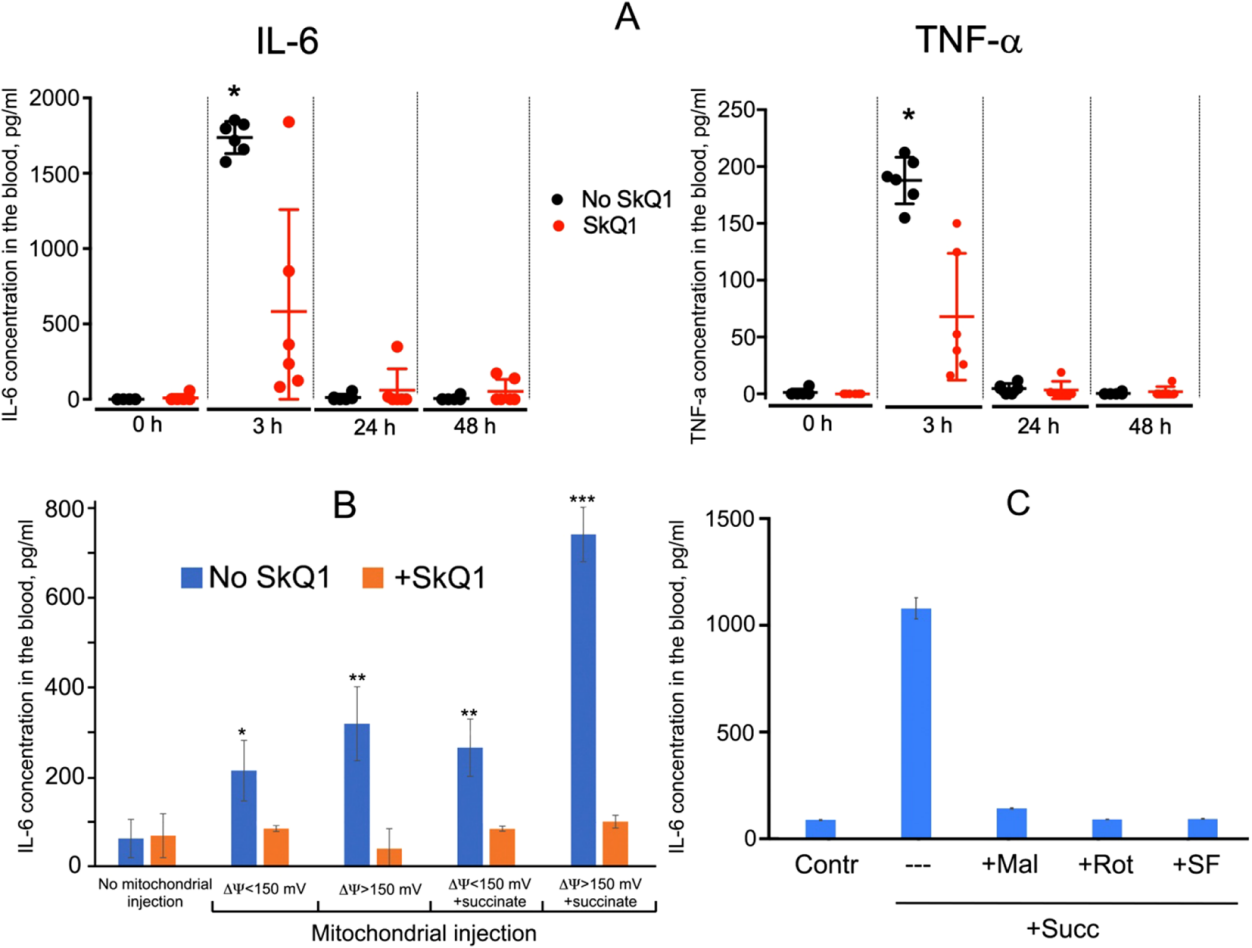
SkQ1 prevents increase in the level of proinflammatory cytokines IL-6 and TNF-α induced by injection of mitochondria. Mitochondria were injected as in Fig. 4. (**A,B**) SkQ1 (1.5 μmol/kg) was administered intraperitoneally daily for 5 days prior to mitochondrial injection. (**B**) Mitochondria isolated as in Fig. 4 and (**A**) were compared with mitochondria isolated in the absence of BSA, which have ΔΨ below 150 mV. Succinate was added to mitochondria prior to injection as in Fig. 4. (**C**) Mitochondria isolated as in Fig. 4 and (**A**) were preincubated with 0.5 mM malonate, 2 μM rotenone or 1 nM SF6847 in the presence of 5 mM succinate prior to injection. Each of the groups contained 6 mice. *p < 0.05. Each of the groups contained 8 mice. In (**A**) the number of mice in each group was 6, and in (**B,C**) in each group 8. *p < 0.05, **p < 0.01, ***p < 0.001.

As with the LPS shock, intravenous injection of mitochondria caused a strong increase of the blood levels of IL-6 and TNF-α (Fig. 5). This effect had a maximum at approximately 3 h after the injection, and was almost completely prevented by SkQ1 (Fig. 5A,B).

Interestingly, addition of 5 mM succinate to the mitochondrial samples prior to the injection strongly enhanced the toxicity and resulted in sudden death of all animals. The mortality was dramatically reduced by SkQ1. Injection of succinate without mitochondria had no effect on mortality (Fig. 4B). In line with increased mortality, addition of succinate to mitochondria prior to the injection strongly stimulated IL-6 production in response to the mitochondria injection (Fig. 5B). These data suggested that mitochondrial respiratory activity played an important role both in production of pro-inflammatory cytokines and in mortality. Indeed, pretreatment of the mitochondria prior to injection with malonate, which inhibits succinate oxidation by Complex II, prevented increase in the level of IL-6 (Fig. 5C). Rotenone, an inhibitor of Complex I, the major producer of mtROS at a high level of membrane potential (ΔΨ)^31^ also suppressed the increase in proinflammatory cytokines (Fig. 5C). Mitochondria used in these experiments were isolated and stored in the presence of 0. 1% bovine serum albumin (BSA) and had a high ΔΨ (more than 150 mV). Notably, a high ΔΨ values were critical for the inflammatory effects, since if BSA was omitted, and ΔΨ in mitochondria did not exceed 150 mV, a significantly lower increase in IL-6 was observed following the mitochondrial injection (Fig. 5B). Most likely, BSA binds free fatty acids that decrease ΔΨ due to H^+^ transportation across the membrane. Consistently, protonophore uncoupler of oxidative phosphorylation CF6847, which reduces ΔΨ prevented succinate-dependent increase in the level of IL-6. These data indicate that production of ROS by Complex I during the energy-dependent reduction of NAD^+^ (the so-called reverse electron transfer) induced by succinate in mitochondria prior to injection is critical for the subsequent production of proinflammatory cytokines in this animal model.

### SkQ1 protects mice from death caused by the cold shock, or by toxic doses of C_12_TPP

**Figure 6:**
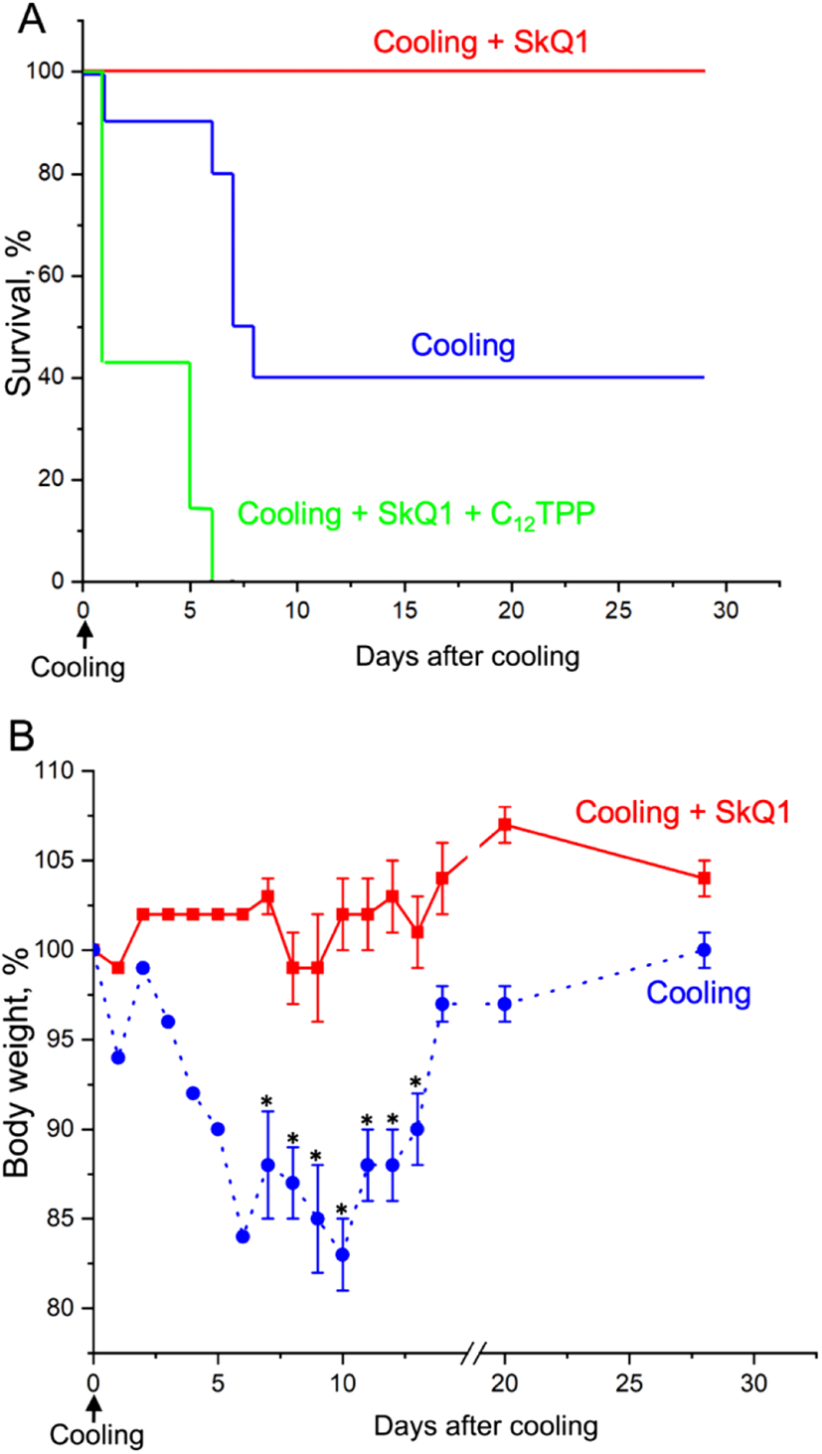
SkQ1 prevents mice from rapid death caused by cooling. Mice were placed at − 20 °C for 1 h and then returned to room temperature. (**A,B**) SkQ1 and C_12_TPP (1.5 μmol/kg) were administered intraperitoneally for 5 days before cooling and for 3 days after cooling. (**B**) Dynamics of mouse body weight after cooling. The group size was 10 mice in experiments with SkQ_1_ or vehicle and 7 mice in the experiments with C_12_TPP.

**Figure 7:**
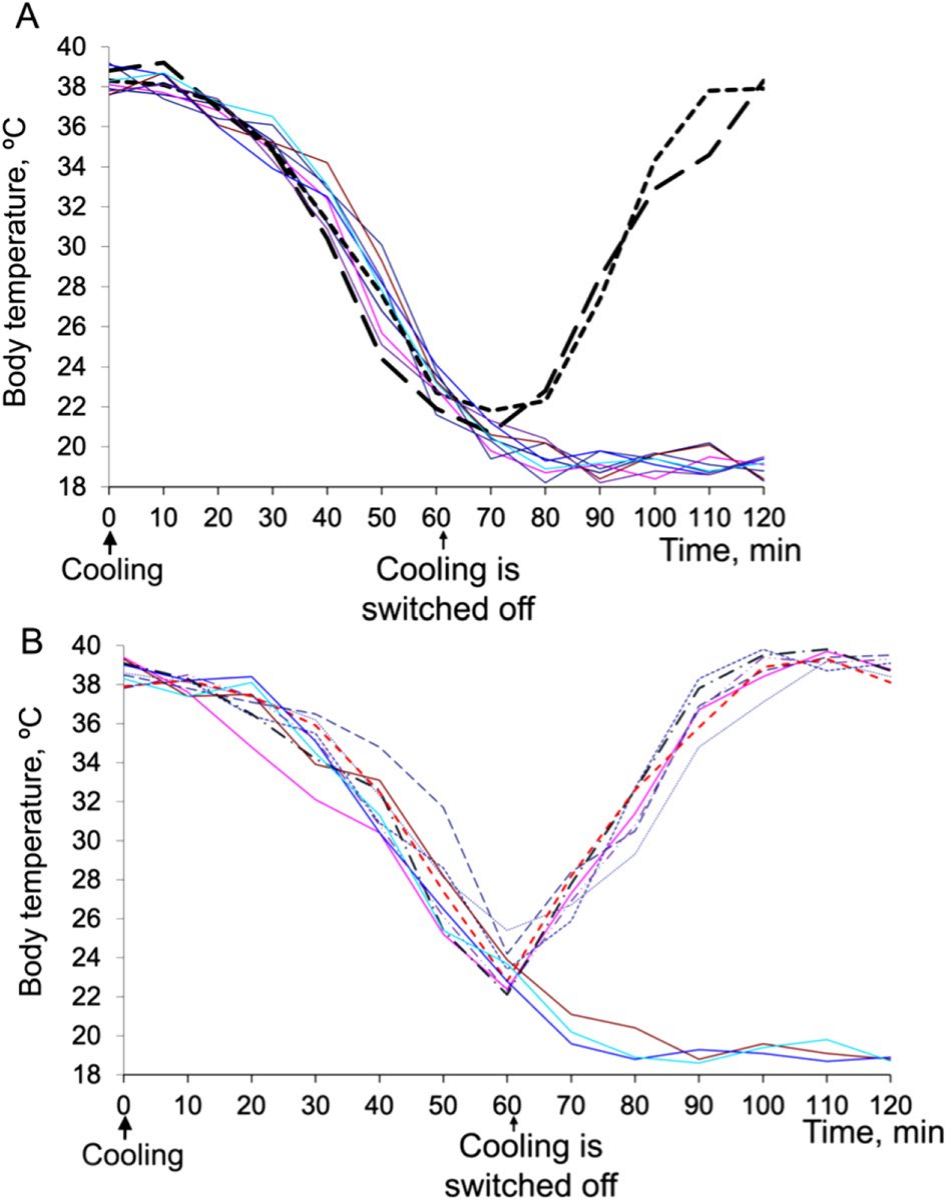
SkQ1, administered 1 min after the end of cold exposure, increased survival and accelerated the return to normal body temperature. Mice were exposed to − 20 °C for 1 h and then returned to room temperature. (**A**) Without SkQ1. (**B**) SkQ1 (1.5 μmol/kg) was introduced intraperitoneally 1 min after cessation of cooling. The solid curves show the body temperature of mice that died after the cold shock (8 out of 10 in (**A**) and 3 out of 10 in (**B**)); dashed curves, mice survived after cooling.

In the next series of experiments (Fig. 6), the rapid death of mice was caused by a completely different shock, namely, by placing the mice for 1 h at − 20 °C. The mortality dramatically increased between the 5th and 8th days after cold shock: the number of survivors dropped to 40%. Pretreatment of mice with SkQ1 completely prevented mortality after cooling (Fig. 6A). The body weight of the survivors decreased and then increased sharply, reaching the initial level after 28 days (Fig. 6B, Table S2). SkQ1 prevented cold-induced loss of body weight. Contrary to SkQ1, pretreatment with similar doses of C_12_TPP increased mortality (in this case, up to 100% by the sixth day after cooling). The introduction of SkQ1 1 min after the end of the cold exposure increased the number of surviving animals (from 2 to 7 out of 10) and slightly increased the rate of rise in body temperature in surviving animals after transferring the mice from the cold to room temperature (Fig. 7).

**Figure 8:**
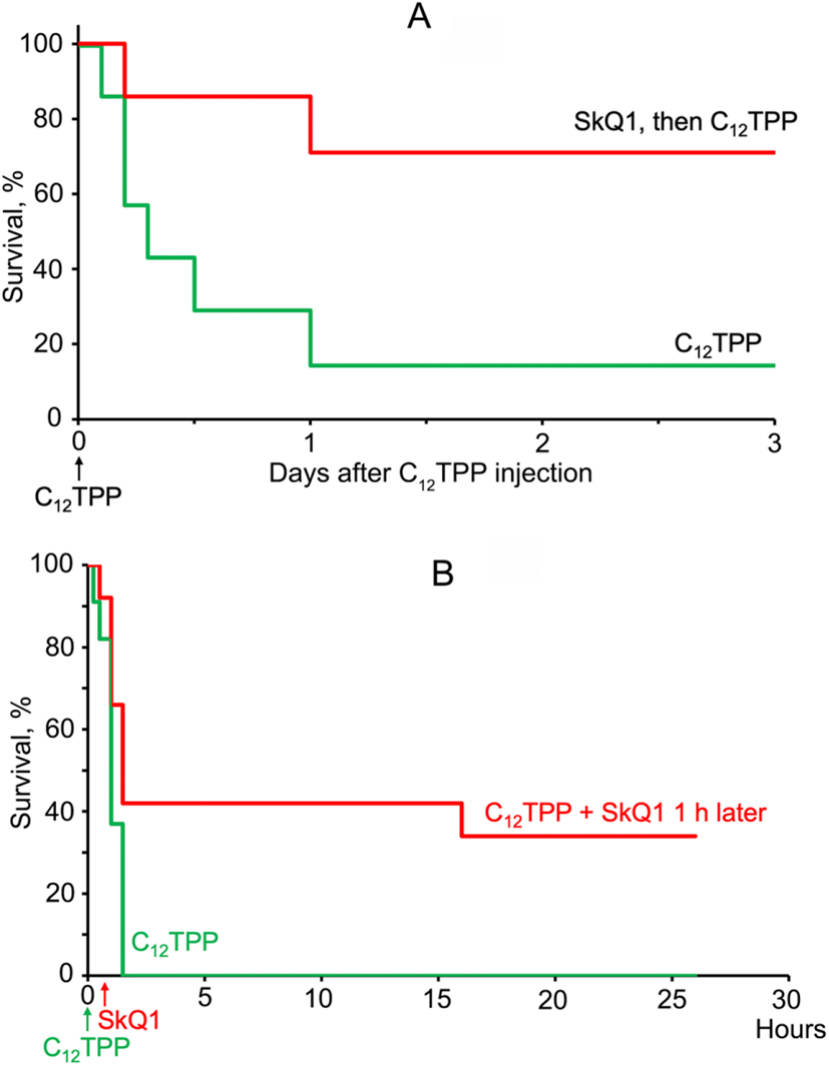
SkQ1 protects mice against toxic doses of C_12_TPP. (**A**) Survival of mice after a single intravenous injection of 34 μmol/kg C_12_TPP. SkQ1 (1.5 μmol/kg/day) was injected as in Fig. 2. The groups contained 7 mice. (**B**) Survival of mice after a single intravenous injection of 42 μmol/kg C_12_TPP. SkQ1 (1.5 μmol/kg) was injected within the first hour after C_12_TPP. Number of mice: 11 with C_12_TPP and 12 with C_12_TPP + SkQ1.

To study an unrelated chemical toxicity shock, we used high doses of C_12_TPP (34 µmol/kg body weight), which resulted in 90% mortality. Five days of pretreatment with SkQ1 (daily intraperitoneal injections of 1.5 µmol SkQ1/kg) decreased the mortality to 30% (Fig. 8A). In the next experiment the dosage of C_12_TPP was increased to 42 μmol/kg All 12 animals died within 1.5 h. When SkQ1 was administered 1 h after C_12_TPP, 1/3 of the animals survived (Fig. 8B).

**Figure 9:**
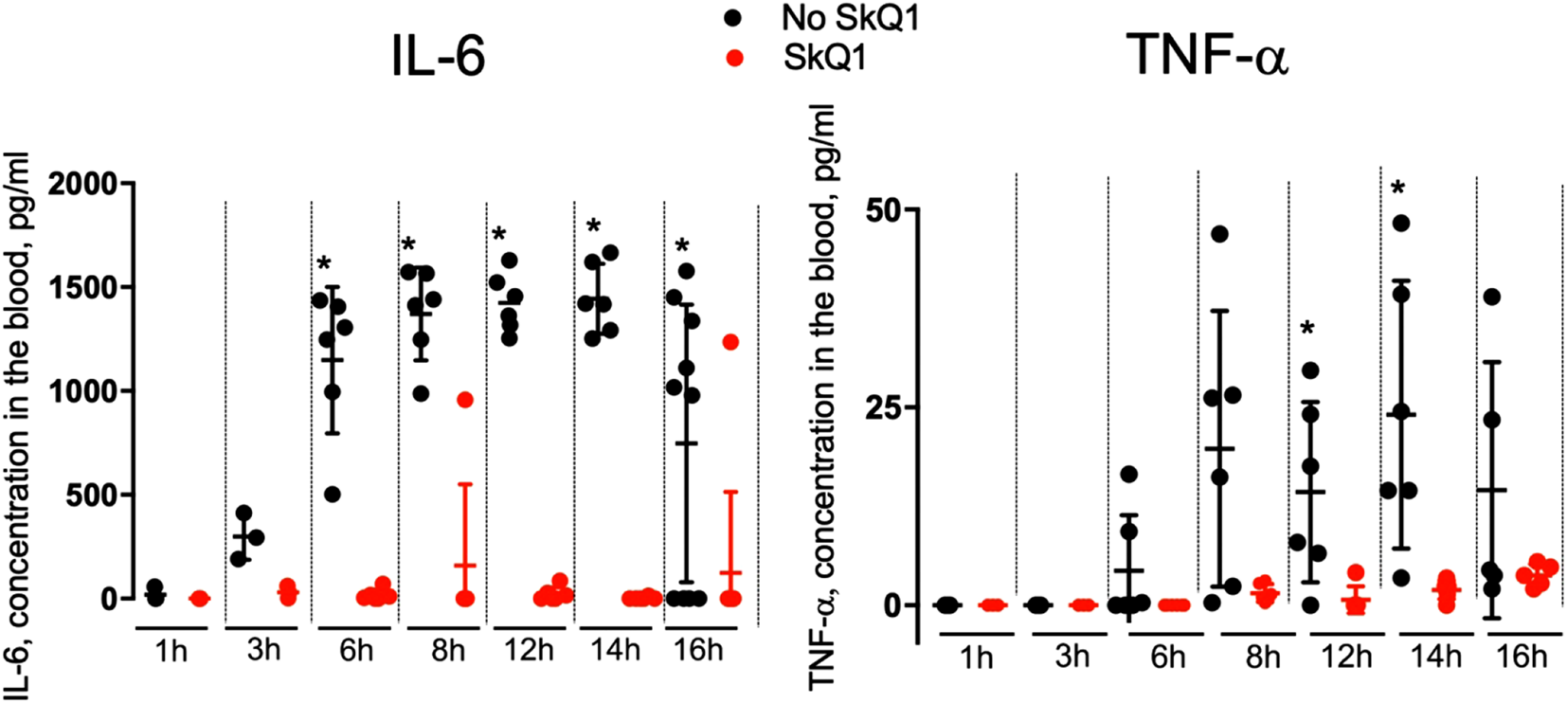
SkQ1 prevents increase in the level of proinflammatory cytokines IL-6 and TNF-α induced by cooling. Mice were placed at − 20 °C for 1 h and then returned to room temperature. SkQ1 (1.5 μmol/kg) was administered intraperitoneally for 5 days before cooling the animals. The group size was 40 mice. *p < 0.05.

**Figure 10:**
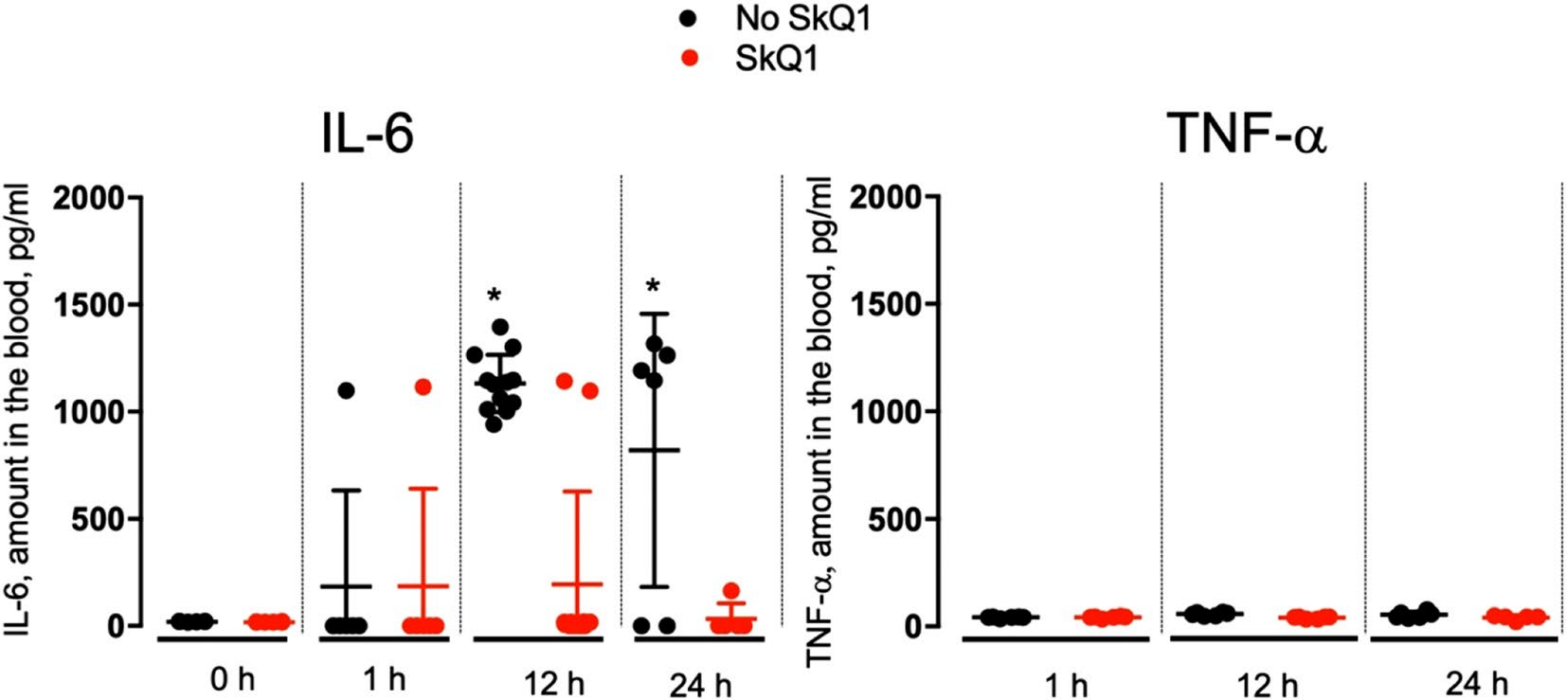
SkQ1 prevents increase in the level of IL-6 induced by toxic dose of C_12_TPP. C_12_TPP (34 µmol/kg) and SkQ1 (1.5 μmol/kg) were injected as in Fig. 8. The group size was 34 mice. *p < 0.05.

Experiments with 1-h cooling of mice at − 20 °C showed a strong increase in the concentration of IL-6 and TNF-α. The peak level of cytokines in the blood began after 6–8 h. The increase in both cytokines was completely prevented by SkQ1 (Fig. 9, Table S5). C_12_TPP-induced toxic shock was also accompanied by an increase in IL-6, while TNF-α levels did not change (Fig. 10, Table S6). Treatment with SkQ1 prevented the C_12_TPP-induced increase in blood IL-6 levels.

The effect of another mitochondria-targeted antioxidant MitoQ was recently analyzed in LPS-challenged mice^32^. MitoQ only slightly reduces the level of pro-inflammatory cytokines in the blood, apparently due to a small (compared to SkQ1) window between the anti- and prooxidant effects of this compound^5^.

## Discussion

### Inhibition of mtROS production prevents death caused by different shocks

In this paper, we demonstrated that rapid deaths of mice after (a) LPS injection, (b) injection of mitochondria into the blood, (c) hypothermia, and (d) injection of the penetrating cation C_12_TPP are prevented by the mitochondria-targeted antioxidant SkQ1. These data further support the idea that neutralization of mtROS by SkQ1 strongly protects organisms from a wide variety of very diverse stressful treatments. Of Indeed, multiple protective effects of SkQ1 and SkQR1 were previously discovered in our group. D.B.Z. and his coworkers described prevention of death caused by ischemia and reperfusion in single-kidney rats^10,14^, by intraperitoneal administration of LPS in newborn rats^30^, and in the rat model of pyelonephritis^33^. More recently the same group^34^ described the protective effect of SkQ1 in single-kidney rats killed by a combination of two shocks: (a) ischemia–reperfusion and (b) intraperitoneal injection of liver mitochondria. B.V.C. and his coworkers showed the protective effect of SkQ1 in mice injected with a lethal dose of TNF-α^35^. Very recently they described prevention of proinflammatory cytokines increase in mice model of ulcerative colitis^36^. A.A.-A. and colleagues observed a favorable therapeutic effect of SkQ1 on experimental autoimmune arthritis induced by collagen injection in specific pathogen-free Wistar rats^37^. Importantly, our current study connects these protective effects to suppression of the pro-inflammatory cytokine production.

The antioxidants that used conjugates of a stable aminoxyl radical 2,2,6,6-tetramethylpiperidin-1-yl)oxyl (TEMPO) as an antioxidant moiety, targeted to mitochondria by conjugation with TPP + (MitoTEMPO)^38^ or hemigramicidin (XJB-5-131)^39^, also suppressed inflammatory response in the murine septic models. Quite recently it was shown that intranasal injection of MitoTEMPO to mice infected with the influenza A virus decreased mortality rate from 50 to 20%^40^.

Some examples of the protective effects of mitochondria-targeted antioxidants (in the majority of cases, SkQ1) are summarized in Table 1. These protective effects have been observed in models of a wide variety of lethal shocks, suggesting that death in all these cases is mediated by mtROS. These results strongly support the concept of programmed death of the organism (phenoptosis) introduced by V.P.S.^28^. Numerous cases have already been collected, confirming the existence and widespread occurrence of this phenomenon in nature^1,41,42^. The most straightforward examples of such a mechanism have been found in unicellular prokaryotes, in which phenoptosis is equivalent to programmed cell death. Both bacteria and archaea have evolved various “abortive infection systems” that can kill the cell, preventing the phage infection from spreading^43–45^. Some of them are based on toxin-antitoxin (TA) modules, which consist of a stable toxin and a short-leaved antitoxin whose expression is blocked by phage infection. Multiple TA modules have been found in bacteria^46^ and more recently in archaea^47^. Another abortive infection systems are associated with retrons, genetic retroelements encoding a reverse transcriptase that produces multi-copy single-stranded DNA^48^. Retrons after phage infection can induce cell death due to the expression of toxins that form pores in the bacterial membrane^49^ or inhibit bacterial growth^50^ thereby preventing the spread of the phage in the population (for details, see Supplementary Discussion 3). Another striking example of phenoptosis in prokaryotes, a phenomenon called “quorum sensing” can also be mediated by toxin-antitoxin modules^51^. This mechanism helps bacteria to maintain their optimal numbers in the environment due to the altruistic suicide of some members of the population.

**Table 1:** Various cases of acute phenoptosis prevented by mitochondria-targeted antioxidants.

No.	Inducers of acute phenoptosis	Antioxidant	References
1.	LPS injection	SkQ1	This article
2.	Intravenous administration of mitochondria (mouse)	SkQ1	This article
3.	Cold stress at − 20 °C for 60 or 90 min (mouse)	SkQ1	This article
4.	Intravenous administration of C_12_TPP (34 μmol/kg body weight, mouse)	SkQ1	This article
5.	Intravenous administration of mitochondria + C_12_TPP (1.5 μmol/kg daily, 5 days, mouse)	SkQ1	This article
6.	Cold stress (60 min) + C_12_TPP (1.5 μmol/kg daily, 5 days, mouse)	SkQ1	This article
7.	Short-term ischemia of single-kidney animal (rat)	SkQ1, SkQR1	Bakeeva et al.^10^ and Skulachev et al.^5^
8.	Short-term ischemia of single-kidney + injection of mitochondria (rat)	SkQ1	Plotnikov et al.^34^
9.	LPS injection (newborn rat)	SkQR1	Plotnikov et al.^30^
10.	Influenza A virus infection (mouse)	MitoTEMPO	To et al. (2020) ^40^
11.	Inflammatory cytokine TNF-a (mouse)	SkQ1	Zakharova et al.^35^
12.	Autoimmune arthritis (rat)	SkQ1	Andreev-Andrievskiy et al.^37^

Among multicellular eucaryotic organisms, the concept of phenoptosis has been confirmed experimentally^52^ and through computer simulations of nematode *Caenorhabditis elegans* populations^52,53^. Ma and colleagues, in genetic screens for mutants sensitive to cold shock, identified a molecular pathway leading to the expression of several genes encoding proteases, which contributed to the programmed death of the organism, which the authors named “stress-induced phenoptosis”^52^. In 2023, the same team^54^ demonstrated that severe freeze–thaw stress in *C. elegans* induces rapid phenoptosis regulated by a signaling cascade dependent on the G-protein coupled receptor (FSHR-1) and the expression of genes involved in proteolysis and lipid remodeling. Importantly, some genes regulated by this signaling are known to be involved in age-dependent pathologies and mortality^55^.

Computer simulations of a clonal population of *C. elegans* under food-limited conditions have shown that life-shortening phenoptosis can improve the adaptive benefits of the colony presumably by reducing food consumption by elderly or sick members^52,53^. Another mathematical model predicted that limiting the lifespan of individuals in a population of *C. elegans* protects against the spread of infection compared to a population of long-lived individuals^56^.

It is reasonable to assume that both animals and humans have developed a phenoptotic mechanism to deal with epidemics. All four of the very different stressors studied above share the ability to induce a significant increase in pro-inflammatory cytokines that is prevented by SkQ1. These results, as well as previously obtained data on the protective effect of SkQ1 in models of inflammatory diseases (see Table 1), indicate that mtROS-dependent activation of innate immunity is involved in the execution of phenoptosis (for details, see Supplementary Discussion and Ref.^57^). With regard to humans, this type of phenoptosis should be viewed as a harmful atavism since we have other ways to limit the spread of epidemics, such as antibiotic and antiviral drugs, quarantine, and vaccination, which does not require a macroorganism suicide.

All four of the very different stressors studied above share the ability to induce a significant increase in pro-inflammatory cytokines that is prevented by SkQ1. These results, as well as previously obtained data on the protective effect of SkQ1 on models of inflammatory diseases (see Table 1), indicate that mtROS-dependent activation of innate immunity is involved in the implementation of phenoptosis (for more details, see Additional Discussion and Ref.^57^). Applied to humans, this type of phenoptosis should be regarded as a harmful atavism, since we have other ways to limit the spread of epidemics, such as antibiotics and antiviral drugs, quarantine and vaccination, that do not require the suicide of the macroorganism.

### The molecular mechanisms of mtROS-mediated acute phenoptosis

Our experiments described above show that the protective effect of SkQ1 is due to its antioxidant properties. C_12_TPP, an analogue of SkQ1, lacking the antioxidant residue, does not prevent mortality under various shock conditions (in fact, it is toxic). It is essential that the therapeutic effects of SkQ1 occur at very low doses of 0.1 µmole/kg^5,10^ or 1.5–2 µmole/kg (this article). An extremely low dose (0.01 µmol/kg) of SkQR1 has been shown to save lives in rats treated with LPS^30^. This high potency of SkQ1 and other mitochondria-targeted antioxidants reflects very strong mitochondrial accumulation and removal of superoxide (O_2_^·−^) or its extremely aggressive derivatives (radicals OH^·^ and HO_2_^·^**)** in the place where they are mainly generated.

**Figure 11:**
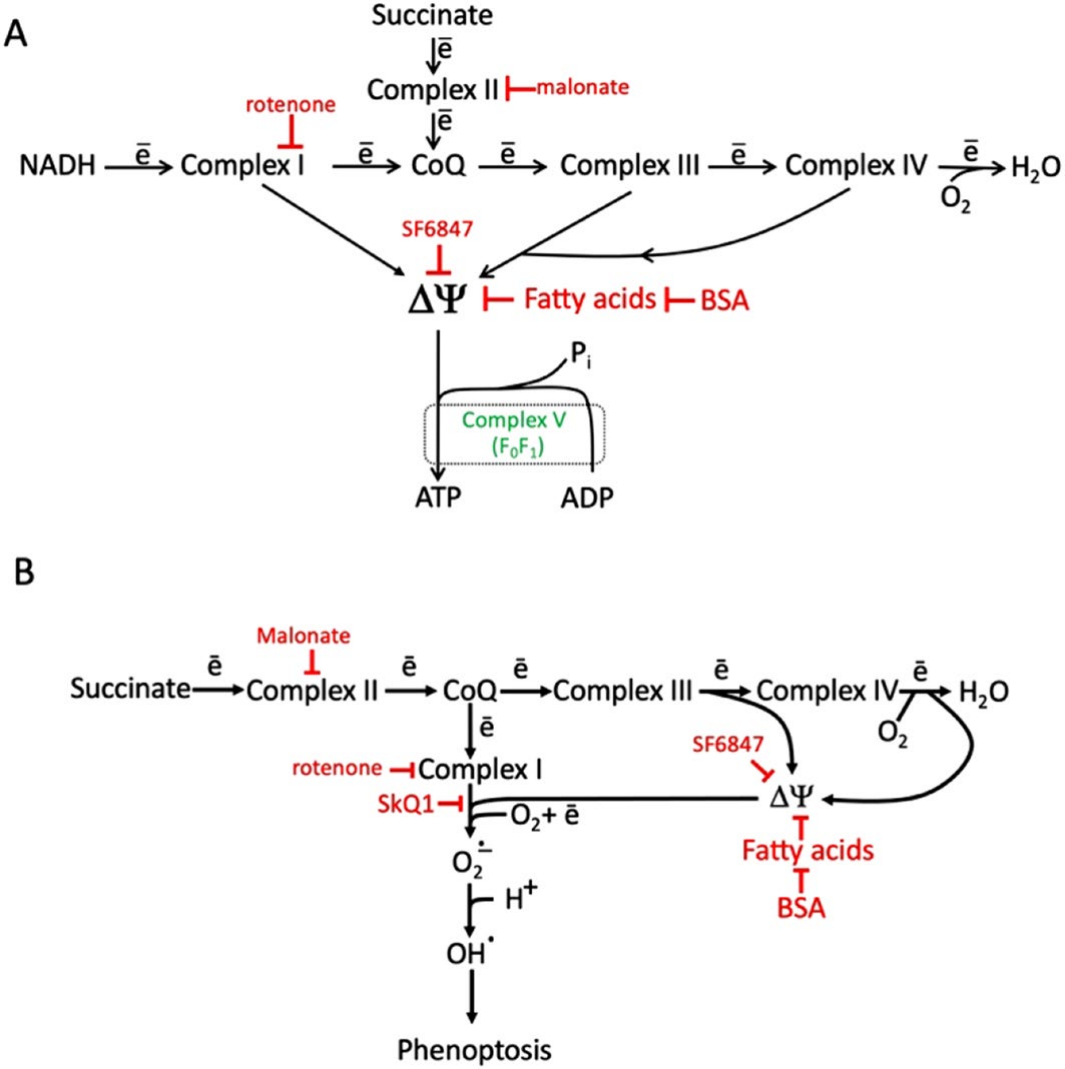
(**A**) Respiratory chains in mitochondria, reducing O_2_ to H_2_O. (**B**) Respiratory chain in mitochondria, reducing O_2_ to H_2_O and superoxide. The specific inhibitors of complexes I and II as are shown in red. Uncoupler SF6847 and fatty acids dissipated membrane potential (ΔΨ). BSA, bovine serum albumin removing fatty acids.

Apparently, respiratory chain complex I is the main mitochondrial enzyme capable of catalyzing the one-electron reduction of molecular oxygen with the formation of superoxide^31,58,59^. This reaction accompanies *reverse* electron transfer (reduction of NAD^+^), which requires a high membrane potential generated by *direct* electron transfer through Complexes II, III and IV. These reactions are shown in Fig. 11 as two alternative electron transfer chains. It is assumed that the electron that reduces O_2_ in Complex I is initially accepted by the iron-sulfur component FeS1a (for a detailed discussion, see^58^). The production of O_2_^·−^ and the subsequent formation of more aggressive HO_2_^·^ and OH^·^ radicals can be used for killing of pathogens^60,61^, but also cause phenoptosis.

It should be emphasized that the source of electrons accepted by the two respiratory chains shown in Fig. 11A,B are NADH and succinate, respectively. The concentration of succinate in resting cells remains very low and may increase in the Krebs cycle during hypoxia^62^. Inflammatory activation of immune cells (caused by LPS and various PAMPs and DAMPs) leads to a rapid increase in succinate concentration due to stimulation of glutamine metabolism or the g-aminobutyrate shunt^63–65^.

In the present study, it was shown that preincubation of isolated mitochondria with succinate increased lethal shock upon injection of mitochondria into the bloodstream (Figs. 4, 5). The effect of succinate may be associated with excessive generation of ROS by reverse electron flow in Complex I, since a decrease in the membrane potential, inhibition of Complex I, and inhibition of succinate oxidation prevented this effect (Fig. 5C). Succinate pretreatment is unlikely to stimulate ROS production by mitochondria after injection (mitochondria rapidly depolarize in the blood due to high Ca^2+^ concentration^66^, but it is likely that ROS-dependent oxidation of mitochondria components *prior* to injection enhanced their pro-inflammatory effect. Alternatively, ROS-producing mitochondria elicited a local inflammatory response at the injection site, which initiated systemic inflammation.

**Figure 12:**
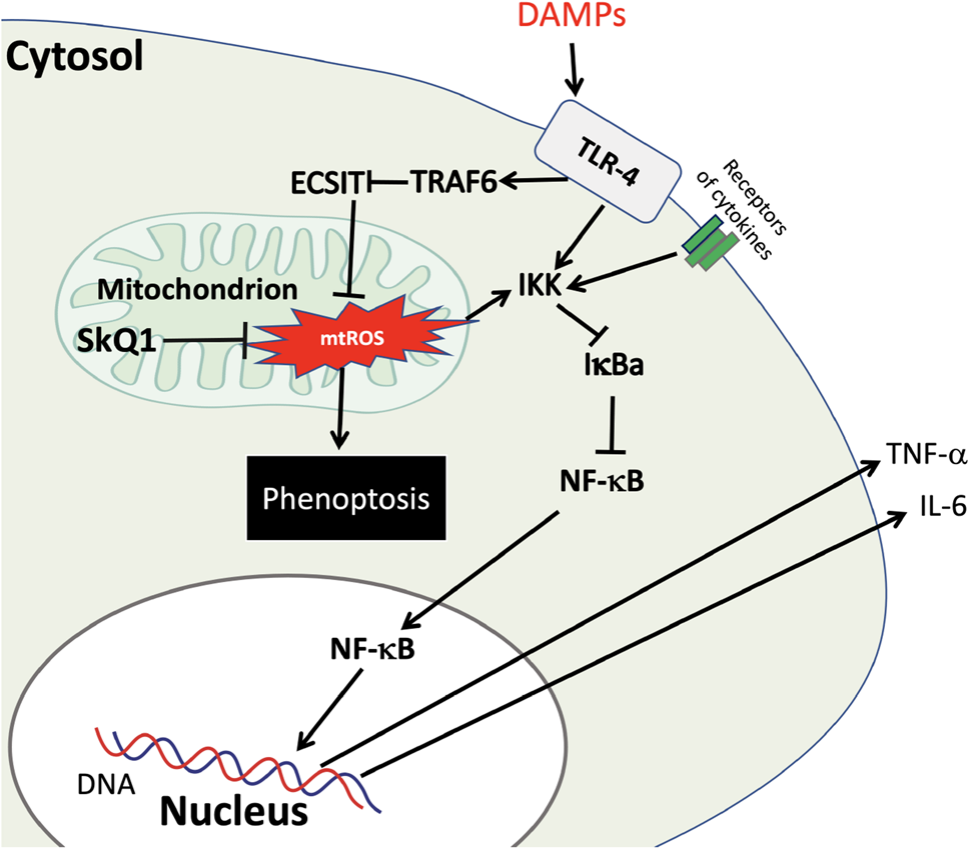
Suggested scheme of initiation of phenoptosis. The details of this scheme are discussed in Supplemental Discussion 1. This figure was made using PowerPoint software (Ver. 16.69).

Superoxide generation by Complex I can also be stimulated by the degradation of ECSIT (evolutionary conserved signaling intermediate of the Toll pathway), which is involved in the assembly of Complex I^61,67,68^. This effect is induced by various PAMPs and DAMPs recognized by Toll-like receptors (such as TLR4)^61,69–82^ as well as by proinflammatory cytokines such as TNF-α and IL-6^71,83,84^ and is mediated by activation of ubiquitin ligase TRAF6 (TNF-α receptor associated with factor 6). TRAF6-dependent ubiquitination of ECSIT leads to its proteasomal degradation and an increase in superoxide production by Complex I. This and some other signaling pathways required for the initiation of phenoptosis are shown in Fig. 12, and some details of this scheme are discussed in the Supplement (Supplementary Discussion 1). The possible role of innate immunity, as well as impairment of barrier function in phenoptosis, are discussed in Supplementary Discussions 2 and 3.

In conclusion, four cases of lethal shoch induced by LPS (PAMP), mitochondria in the blood (DAMPs), short-term cooling, or a toxic compound (C_12_TPP) were efficiently prevented by mitochondria-targeted antioxidant (SkQ1) indicating that here we address a general mechanism of organismal programmed suicide (phenoptosis). The above logic suggests that SkQ1 can be used to save the life of patients in critical conditions of a very different origin. In particular, SkQ1 can be applied for therapy of sepsis or systemic inflammatory response syndrome (SIRS) caused by massive trauma, burns, or major surgery.

## Methods

### Regulatory statement

All methods of study were performed in accordance with the relevant guidelines and regulations.

### Compounds and reagents

SkQ1 and C_12_ TPP were synthesized according to previously published methods^20^ and stored at + 2 °C in the dark. Stock solutions contain 2.8 mM SkQ1 or C_12_ TPP, propylene glycol (500 mg/ml) and lactic acid (2.9 mg/ml). This solution without SkQ1 or C_12_ TPP was used as placebo stock. Solutions were pyrogen-free and sterile. These stock solutions were diluted 7 times with sterile 0.9% NaCl no more than 2 h before administration to animals. All other reagents used in this work, except those specifically indicated, were obtained from Sigma, USA.

### Laboratory animals and their handling

The experiments were approved by the Commission on Bioethics of the Institute of Mitoengineering of Moscow State University (Protocols 179, 183 and 187) and by the Institutional Ethics Committee of the A. N. Belozersky Institute of Moscow State University (Protocol 7, 2019). C57Bl/6J line laboratory mice were obtained from the Center for Genetic Resources of Laboratory Animals of the ICG SB RAS (Novosibirsk, Russia). Mice (3–28 months old, 483 in total) were kept by five animals per individually ventilated cage (IVC system, TECNIPLASTS.pA, Italy) with free access to food (granulated autoclavable feed produced by Sniff Spezialdiäten GmbH, Germany) and water purified by reverse-osmose with help of a custom-made system produced by “Median Filter LTD”, Russia. Housing conditions were free from specific pathogens according to FELASA guidelines (health monitoring was performed by qPCR-RT and ELISA at BBTLAB, Novosibirsk, Russia), with a light mode of 12/12 (light on at 09:00); the air exchange rate in the rooms was at least 15 rev/h, with an air temperature of 20–24 °C and a humidity of 30–70%. Lignocel wood chips (JRS, Germany) were used for cage flooring.

Assignment of animals into experimental and control groups was carried out randomly, using weight as a criterion so that the average weight of the groups would be equal. This study was performed in accordance with ARRIVE guidelines (https://arriveguidelines.org).

The animals that survived after the shocks were monitored for: temperature (measured with an infrared thermometer), ruffled coat, hunchback, swelling of the abdominal cavity, discharge from the eyes and nose, and weight dynamics.

### Administration of SkQ1 and LPS to mice

SkQ1 or C_12_TPP (see Fig. 1) were administered to the mice intraperitoneally after dilution of a concentrated stock solution as described above at a dose of 1.5 μmol per kg of animal weight once a day for 5 consequent days, with the last injection made 12–16 h before the challenge with LPS, or 1–2 h before the other shocks.

Lipopolysaccharide (LPS) from *E. coli* O111:B4 at doses 10–40 mg/kg was injected intravenously.

### Intravenous administration of isolated mitochondria

For the mouse liver mitochondria isolation protocol, see the Supplement. Where indicated BSA was omitted from isolation and storage medium.

The mitochondrial ΔΨ was measured by monitoring the changes in the fluorescence of safranin O (final concentration, 4.3 μM) at excitation/emission wavelengths of 485/586 nm using a Cary Eclipse fluorescence spectrophotometer (Agilent Technologies, USA) and calibrated using a K+ gradient precisely as described in Ref.^13^.

Just before a single administration into the tail vein, isolated mitochondria were diluted with a 0.9% solution of NaCl to a final protein concentration of 2 mg/ml and homogenized in a Potter microhomogenizer (by 12 passes for 1 min at 4 °C). This suspension was supplemented with 5 mM succinate or the inhibitors as shown in Fig. 5C. Mitochondria were administered at the rate of 10 mg of protein/kg of body weight. Surviving mice were treated with SkQ1 or vehicle for up to 15 days after mitochondrial injection and their weight was monitored.

### Exposure of animals to cold stress

Animals were exposed to low temperatures (− 20 °C) for 60 min. The body temperatures of the surviving animals were determined every 10 min (see Suppl. Methods). The ability of the animal to maintain a sitting position was monitored. The weight of the animals was measured daily after the end of the cold exposure using a Pioneer 2102 electronic scale (USA) with an error of 0.05 g.

### Measurements of TNF-α and IL-6

In the blood plasma of the mice, the cytokine content was determined by enzyme-linked immunosorbent assay (ELISA) with mouse TNFa DuoSet and mouse IL-6 DuoSet (R&D Systems, USA) using a Hospitex Diagnostics plate spectrophotometer reader. For details Supplemental Methods.

### Statistical analysis

The data were analyzed using Excel (Microsoft, USA) and Prism (GraphPad, USA) software. The t-test was used to assess related samples, and the nonparametric Mann–Whitney test was used for unrelated samples. The data obtained are presented as the mean ± standard deviation. The differences were considered to be significant at p < 0.05.

## Supplementary Information

Supplementary Information.

## Data Availability

The datasets and raw data used and/or analyzed during the current study are available from the corresponding author on reasonable request.
