# Supplementary material for: Mitochondrion-targeted antioxidant SkQ1 prevents rapid animal death caused by highly diverse shocks
Source: Sci Rep. 2023 Mar 15;13:4326. doi: 10.1038/s41598-023-31281-9 (PMC10017827; doi:10.1038/s41598-023-31281-9)
Supplement: Supplementary file 1 — Supplementary Information. [file 41598_2023_31281_MOESM1_ESM.docx]

**Supplemental Information**

**Methods**

**Supplemental Discussion 1.**

**Discovery of** **specific messengers of acute phenoptosis**

The scheme presented in Fig. 12 implies DAMPs as the *messengers of acute phenoptosis*. What molecules could they be? In 1997, J. Fujita and colleagues**^1^** reported the discovery of an RNA-binding protein composed of 172 amino acid residues. First, it proved to be inducible in mice or humans by cooling. The protein and its mRNA concentrations strongly increased when the body temperature decreased to 32 °C. The protein was called “Cold Inducible RNA-binding Protein” (CIRP). Later, Liu and others **^2^** exposed mice to a 4 °C temperature for several hours. The authors suggested that CIRP prolonged animal survival during cold exposure. However, it seems that the authors failed to distinguish between the cause and its effect. They only observed a strong increase in CIRP concentration in cold-exposed animals. Common biological logic suggests that CIRP is induced to protect animals from cold shock. However, the phenoptosis concept turns the whole picture upside down. What if CIRP overexpression is intended not to protect the organism but is in fact a cue for biochemical suicide? It was later reported by several other groups that the death of organisms during many different shocks (including cold shock) is accompanied by a manifold increase in CIRP levels in the blood and tissues. Finally, animals die. In strong favor of this logic, in CIRP*^-1-^* animals, the lethal effect of cold shock proved to be much smaller than that in wild-type animals **^3,4^**.

Normally, CIRP operates as a chaperone normalizing mRNA conformation. Under normal conditions, CIRP is localized in the nucleus **^3,5^**. Cooling and other shocks were found to induce (i) a many-fold increase in CIRP due to activation of the CIRP gene by NF-κB (see Fig. 12), (ii) CIRP accumulation in special cytosolic membrane vesicles **^6^**, (iii) attachment of these vesicles to the outer cell membrane and (iv) extrusion of CIRP from the vesicles to the extracellular space **^3,6^**. The following shocks were shown to initiate the above-listed events: injection of mitochondria into the blood, hypoxia/reperfusion, active acute damage to the lung, kidney, pancreatitis, arthritis, and some other shocks **^3,7-14^**. In CIRP*^-1-^* mice, all these shocks were arrested, preventing death. Injection of recombinant CIRP to CIRP*^-1-^* animals stimulated shock development **^3,4^**.

The next step consists of the binding of extracellular CIRP by TLR. The binding induces activation of ubiquitin ligase TRAF6 (TNF-α receptor associated with factor 6)**^15,16^**. TRAF6 ubiquitinates ECSIT (evolutionarily conserved signal intermediate of the Toll pathway) initiating its proteasomal degradation **^16-18^**. ECSIT is involved in assembly of respiratory chain Complex I? so its degradation results in production of large amounts of O_2_^•-^ from O_2_ by Complex I*.* Another consequences of TLR activation is ROS-dependent activation of IκB kinase (IKK) **^16,19-21^**. IκB is an inhibitor subunit of transcription factor NFκB and its phosphorylation followed by proteasomal degradation results in activation of NFκB. NFκB controls expression of various proinflammatory cytokines.

It is interesting that CIRP is not the only protein operating as a messenger of acute phenoptosis. In fact, a similar function is inherent to so-called high-mobility group box 1 (HMGB1) **^22^**. This protein, like CIRP, is normally localized in the nucleus, operating as a nucleic acid chaperone (in contrast to CIRP, dealing with RNA, HMGB1 operates as a DNA сhaperone) **^7,23-25^**. Again, similar to CIRP, HMGB1 is recruited when shock-induced acute phenoptosis is switched on. Among such shocks, there are hepatic ischemia/reperfusion **^24,26^**, hemorrhagic shock, sepsis **^23^,** cytokine storm **^24^,** etc. To stimulate a shock, the HMGB1 concentration is strongly increased. The protein escapes the nucleus and cytosol and is transported from the cell to the extracellular space by special vesicles appearing in the cytosol **^7,23,25,27^**.

It is hardly surprising that a long list of the same functions required for acute phenoptosis includes two different proteins, i.e., CIRP and HMGB1. Phenoptosis is a process that is counterproductive for the individual, similar to some other death-associated altruistic events. This feature makes mutation in a *single* gene involved in phenoptosis look favorable from the point of view of an individual. However, if phenoptosis can be realized by two parallel metabolic pathways, mutations in *two* genes are required to prevent this process. This means that the probability of phenoptosis should increase, lowering the probability of the spread of an epidemic.

The last point to be considered in this paragraph is the names of two phenoptosis messengers, i.e., *cold-inducible RNA-binding protein* (CIRP) and *high mobility group Box 1* (HMGB1). For the first one, both terms, cold-inducible and RNA-binding, are misleading if we consider the pro-phenoptotic effect of CIRP. The protein is involved not only in cold shock but also in numerous other shocks, and there is no reason to include “cooling” in the name of the protein. RNA binding by CIRP is related to “civil” CIRP function and not to the excess CIRP recruited to kill the organism.

The name of the second messenger (HMGB1) is apparently related just to a specific event of the discovery of this protein rather than to its “civil” or “military” functions. Taking into account the above reasoning, we suggest to nominate the first and second messengers as (a) *phenoptosis messenger 1* (*PM1*, former CIRP) and (b) *phenoptosis messenger 2* (*PM2*, former HMGB1).

The final result of signaling pathways induced by DAMPs (including PM1 and PM2) (Fig. 12) can be lethal for the organism. At the same time, such biochemical suicide prevents epidemics and pandemics by eliminating those individuals who failed to resist the infection and, hence, have become a source of the infection. As we mentioned above, modern humanity has other and not so cruel means to fight epidemics: anti-infection drugs (antibiotics/antimycotics/antivirals), quarantine, and vaccination. They allow us to consider the suicide mechanism as an evolutionary atavism that we inherited from our animal ancestors. In light of this concept, a promising universal approach to saving the life of patients is suggested: to inhibit phenoptosis by artificial compounds that interrupt some of the Fig. 12 pathways. At present, we may already try to use three different inhibitory mechanisms: (a) inhibition by antibodies of proteins involved in the suicide scheme (in fact, this approach is already applied by the treatment of COVID-19 patients with anti-cytokine antibody-based drugs) **^28^**, (b) injection of short peptides identical to some parts of the amino acid sequences of PM1 (CIRP) **^2^** or PM2 (HMGB1) **^29^**, and (c) injection of mitochondrial antioxidant SkQ1 preventing respiratory storm, mROS burst, and the consequent events of the suicide scheme. The storm in question can be arrested if the PM1-binding domain of TLR facing the extracellular outer cell membrane surface is blocked by a synthetic peptide composed of 16 amino acid residues identical to the middle part of PM1. The peptide in question was called C23 and was shown to bind with the TLR platform, preventing PM1 interaction with TLR **^3,7,30^**. The same effect is also produced by a 7 amino acid peptide called M3 **^3^**. A similar approach can be used for another phenoptosis messenger, PM2. Four amino acid synthetic peptide, P5779, was shown to inhibit cytokine storms induced by PM2 **^29^**. In 2020, some indications that the phenoptosis messenger PM2 acts as a mediator of severe lung inflammation in COVID-19 were reported. ^69-71^.

Shortly before sending our manuscript to the publishers, an important paper was published by I.A. Pomytkin and coworkers ^72^. It was shown that interaction of PM2 with TLR 4 is necessary for infection in the death of mice treated with LPS. Death was completely prevented by a peptide agonist of *δ*-opioid receptors called “leytragin", which inhibits PM2 secretion (Fig.S1)^72^.

**Supplemental Discussion 2.**

**Innate immunity as the executioner of phenoptosis – suicidal** **programmed death of an individuals**

The concept of phenoptosis as the programmed death of individuals was introduced by V.P.S. 25 years ago **^31,32^** and has been significantly developed in recent years **^33^**. It was based on the conclusion that altruistic programmed death of individuals can be a subject of Darwinian selection that contributes to inclusive fitness. In bacteria, phenoptosis (another name, "abortive infection system") evolves as an adaptive defense strategy against phage infection. In humans, activation of innate immunity in response to viral and bacterial infections is often responsible for a severe course of the disease. This may reflect the action of a suicidal defense strategy against the spread of pathogens rather than an aberrant overstimulation of defense responses. In the pathogenesis of sepsis, massive trauma (including surgery and burns), ischemia/reperfusion, and some toxic insults, the immune response is the leading cause of mortality. We hypothesize that innate immunity can be evolutionary and functionally related to phenoptosis, which acts as an executioner mechanism of a suicidal program.

The basic principle of the innate immune system, formulated by C. A. Janeway Jr **^34^**, states that invading pathogens are recognized due to a limited number of products, termed pathogen-associated molecular patterns (PAMPs), which are conserved among viruses and diverse groups of microorganisms, including nonpathogenic ones. PAMPs are recognized by pattern recognition receptors (PRRs). The first of the discovered PRRs are Toll-like receptors (TLRs). In humans, up to ten such PRRs are described **^35^.** In addition to PAMPs, the same TLRs, as well as some other PRRs, recognize endogenous molecules released from damaged cells (damage-associated molecular patterns, DAMPs). Today, a large number of DAMPs of various chemical natures and origins have been identified, and this list is expanding rapidly. Various types of DAMPs and their role in inflammation have been extensively reviewed **^36^**.

The release of DAMPs has been recognized as a critical event in the pathogenesis of sepsis, various viral (including COVID-19), bacterial infections, and systemic sterile inflammation, which is an inevitable consequence of massive trauma (including surgery), burns, hemorrhagic shock, ischemia/reperfusion injury and some toxic insults. DAMPs stimulate inflammatory cytokine production, pyroptosis, and immunothrombosis, contributing to poor outcome of these pathologies. Genetic knockouts, neutralizing antibodies, and some pharmacological agents that significantly inhibit DAMPs or prevent the activation of DAMP receptors may reduce mortality in animal models of sepsis **^37^** and systemic sterile inflammation **^38^**.

Host-pathogen interactions are the main driving force behind the evolution of immunity **^39^** and phenoptosis **^32^**. Mathematical modeling demonstrates that individual suicide can be an adaptive host defense strategy in an infected population that satisfies two conditions: (i) a spatially structured environment that limits free mixing within the population and (ii) an extremely dangerous pathogen with a very high transmission rate **^40^**. These predictions were experimentally verified by Refard and Kuemmerli in a model of *Escherichia coli* infected with λ-phage **^41^**. It was shown that *E. coli* strain bearing an "abortive infection system" based on the RexA/RexB module, which stops cell division upon λ-phage infection, outcompeted the Rex-negative strain in a "structured medium" (it was enough to add 1.5% agar to the medium to limit the spread of bacteria) **^41^**.

The discovery of CRISPR-Cas systems in archaea and bacteria demonstrates that adaptive immunity already appears in prokaryotes. CRISPR (short palindromic repeats grouped at regular intervals) is a collection of sequences in the genome of prokaryotes derived from the genomes of phages that have previously infected the host. They help to recognize DNA of similar phages upon infection and destroy it using Cas endonucleases. According to Koonin and Zhang **^39^**, genomic loci encoding CRISPR-Cas systems are colocalized with loci encoding some abortive infection systems (toxin-antitoxin modules) and restriction-modification systems, which implies an evolutionary and functional relationship between the immune system and the altruistic suicidal strategy.

One of the purposes of PAMP and DAMP sensing is to attract immune cells to the sites of infection or injury to fight pathogens and to facilitate tissue repair and regeneration. Traditionally, the killing effects of innate immunity are associated with aberrant overstimulation. However, according to our hypothesis, they may reflect the action of a suicidal defense strategy against the spread of pathogens. In the case of noninfectious diseases, the significance of phenoptosis is probably associated with severe neurological disorders that often accompany systemic inflammation. Sterile systemic inflammation and styryl sepsis are often associated with encephalopathy which manifests itself under conditions ranging from delirium to coma. DAMP-dependent signaling has been shown to be responsible for encephalopathy caused by hepatic failure **^42^** and cerebral ischemia **^43^**. Importantly, in severe respiratory viral infections, such as COVID-19, encephalopathy, delirium and long-term cognitive impairment are correlated with systemic inflammation **^44^**. Most likely, in severe pathologies, phenoptosis is activated to cleanse the population of dangerous individuals with physical and mental disabilities. Fighting this atavistic program can become the most important task of modern medicine.

Mitochondria not only supply a significant portion of DAMPs but, more importantly, also control the main signaling pathways involved in the activation of innate immunity and inflammation **^45,46^**. Mitochondrial reactive oxygen species (mtROS) are responsible for the activation of NADPH oxidase in neutrophils **^47^** and NET release **^48^**, important components of antimicrobial defense. mROS are involved in the activation of the NLRP3 inflammasome in macrophages **^49^** and thus in the production of inflammatory cytokines. Moreover, mtROS are critical for cell damage and control some forms of programmed cell death (pyroptosis and NETosis), leading to the release of DAMPs.

The mitochondria-targeted antioxidants developed in our laboratory (SkQs) suppressed the manifestations of brain damage, myocardial infarction, and kidney injury induced by ischemia **^50^**. These antioxidants were the first to show a decrease in mortality in the kidney ischemia/reperfusion model **^50^**, in models of pyelonephritis **^51^** and neonatal endotoxemia **^52^**, as well as in a murine model of systemic inflammation induced by intravenous injection of TNF **^53^**. The therapeutic effects of mitochondria-targeted antioxidants in such a variety of severe pathologies can be explained by the suppression of phenoptosis mediated by systemic inflammation.

In the initial concept of phenoptosis, V.P.S. suggested that aging can be described as chronic (slow) phenoptosis and that mROS play a key role in senile pathologies and in “healthy aging” **^31,32^**. In support of this hypothesis, mitochondria-targeted antioxidants (SkQs) have been shown to significantly increase the lifespan in various animal models **^54^**. These data suggest that the stimulation of innate immunity associated with mROS production is an execution mechanism of late aging (aging-linked acute phenoptosis).

The close link between the immune system and aging has been confirmed in genetic studies of longevity in *Drosophila melanogaster*. In three independent studies **^55-57^**, long-term (up to 35 years) selection of flies for longevity and subsequent analysis of genomes revealed important changes in the innate immunity genes. Moreover, conditional knockdown of the Toll receptor in adulthood using *in vivo* RNA interference (RNAi) significantly increased the lifespan of the flies **^57^**. In other studies, RNAi-mediated suppression of inflammatory NF-κB signaling in glia **^58^** or the secretion of antimicrobial peptides **^59^** led to an increase in the lifespan of *D. melanogaster* by more than 60%.

An experiment similar to that described above with *D. melanogaster* was realized in nature during the evolution of bats. Bats are known to be a viral reservoir containing more pathogens than any other mammalian species. The unique mechanisms of immune tolerance allow bats to coexist peacefully with various pathogenic viruses, including coronaviruses **^60-62^**. Bats have a strong interferon response to viral RNA but reduced sensitivity to viral DNA. In part, this reflects the absence (in various bat species) of the PYHIN gene family, which includes cytoplasmic DNA sensors (AIM2 and IFI16) capable of activating the inflammasome and interferon pathways. In addition, bats have a mutated mechanism (STING/cGAS) responsible for DNA-dependent interferon activation. Moreover, the NLRP3 inflammasome, which is a key sensor for various pathogens, is suppressed at several levels, including mutations in caspase-1 **^63^**. In some bat species, additional mechanisms of immune tolerance have been described, such as upregulation of anti-inflammatory IL-10 expression and downregulation of TNF-α expression **^60^**.

Bats are the only flying mammals, and it is likely that immune tolerance evolves as an adaptation to active metabolism and muscle work, which are accompanied by an increase in body temperature and the release of DAMPs **^62^**. It is important to note that the lifespans of bats are significantly longer than those of other mammals of the same size. Typically, bats have a maximum lifespan of approximately 20 years, while some species of bats (such as Brandt's bat, little brown bat, etc.) have a maximum lifespan of 30-40 years **^60^**. It can be assumed that the development of immune tolerance in bats suppresses phenoptosis which is responsible for aging.

A significant body of evidence indicates that low-grade inflammation in humans not only accompanies old age but is also one of the main drivers of aging **^64^**. Our hypothesis suggests that suppression of innate immunity (for example, with mitochondria-targeted antioxidants) may be a promising strategy not only in the therapy of various diseases but also in the fight against aging.

**Supplemental Discussion 3**

**Impairment of barrier function as a universal tool of phenoptosis**

Phenoptosis can be especially effective against the spread of infection in a population. This is well illustrated by a recently discovered mechanism of retron-mediated programmed death of bacteria as their tool against bacteriophages ^65^. In this paper, Millman *et al.* discovered the mechanism by which bacterial retrons (including Ec48 retron) provide an effective defense against different phages. Retrons are bizarre genetic elements consisting of a noncoding RNA and reverse transcriptase that generate chimeric RNA-DNA duplexes when activated. Millman *et al.* found that Ec48 retron is activated when a phage inhibits RecBCD, the core antiphage system of a bacterial cell. The authors suggested that Ec48 retron constantly monitors the integrity of the RecBCD complex which is compromised by phage inhibitors. Activated Ec48 retron, in turn, stimulates toxic transmembrane effector proteins that impair bacterial cell membrane integrity, causing phage-infected bacteria to die before the phage completes its replication cycle. It was experimentally demonstrated that this defense mechanism effectively aborts phage infection by the programmed death mechanism, i.e., bacterial phenoptosis. In this case, the critical element of the phenoptosis mechanism is a disruption of the bacterial membrane barrier. Interestingly, impairment of some barriers critical for living is apparently used in the phenoptosis of animals, namely, systemic inflammatory response syndrome (SIRS) and septic shock. Excessive inflammatory reactions in these conditions are associated with high levels of circulatory cytokines and production of matrix metalloproteinases (MMPs) in endothelial cells of blood vessels, an effect leading to the cleavage of cell-to-cell contacts. Disruption of the contacts violates the integrity of the endothelium and leads to increased permeability of blood vessels for both plasma and inflammatory cells **^66^**. This, in turn, causes swelling, organ dysfunction and finally death of the patient **^67,68^**. Notably, this type of phenoptosis is apparently mROS-dependent since inflammation-driven endothelial permeability is inhibited by SkQ1 **^53^**.

**Supplemental methods.**

**Mitochondria isolation and characterization**

Mitochondria were obtained by a standard method as described by Hoogeboom **^73^**. Mouse liver fractionation was performed by differential centrifugation of a homogenate obtained with the help of a Potter homogenizer (clearance 150 µm) in a solution of 250 mM sucrose with pH 7.4 (HEPES-NaOH) containing 0.1% albumin and 0.5 mM EGTA in a ratio of 10/1 (v/w) at 4 °C. Centrifugation parameters - 600 g, 10000 g, 10 minutes, 4 °C. The resulting mitochondrial suspension was stored on ice at 0-4 °C and used for 1-2 hours. To assess the functional state of the isolated mitochondria, the rate of oxygen consumption was measured at 25 °C using a closed-type Clark electrode on a Hansatech oxygraph (Great Britain) as described previously **^74^**. Mitochondria (0.05-0.1 mg protein) were incubated in an oxygraph cell containing 0.5 ml of MIR05 respiration medium (EGTA 0.5 mM, 3 mM MgCl_2_ * 6H_2_O, 60 mM potassium lactobionate, 20 mM taurine, 10 mM KH_2_PO_4_, 20 mM HEPES, 110 mM sucrose, 1 g/L BSA), and the efficiency of respiration was evaluated in the presence of 5 mM succinate/2 μM rotenone. The ratio of the maximal rate of coupled respiration in the presence of 1 µM oligomycin to the maximal rate of uncoupled respiration (respiratory control ratio, RCR) in the presence of 0.1 mM ADP or 10 nM FCCP was calculated, and mitochondria with an RCR of at least 5 were used. The protein content of the mitochondrial suspension was determined by the bicinchoninic acid method in accordance with the manufacturer's recommendations; a 1 mg/ml solution of bovine serum albumin was used as a standard. The mitochondrial membrane potential (ΔΨ) was measured precisely as described earlier in **^13^**. Fluorescence of safranin O (final concentration, 4.3 μM) was measured at excitation/emission wavelengths of 485/586 nm using a Cary Eclipse fluorescence spectrophotometer (Agilent Technologies, USA). ΔΨ was calibrated using a K+ gradient as described in **^75^**. Hydrogen peroxide production by the mitochondria was estimated using the method described in **^76^**.

**Determination of body temperature of mice**

The measurement of body temperature in the groin of the mouse was carried out using an infrared sensor TF-600 PCT (China) with an error of 0.2 °C **^77^**. Three measurements were made with an interval of 5 seconds, and the mean and standard deviations were calculated.

**Determination of cytokine levels in blood plasma**

In the blood plasma of the mice, the cytokine content was determined by enzyme-linked immunosorbent assay (ELISA) with mouse TNFα DuoSet and mouse IL-6 DuoSet (R&D Systems, USA) using a Hospitex Diagnostics plate spectrophotometer reader.

Blood samples from isoflurane-euthanized mice were taken from the right ventricle with a 2-ml injection syringe with a G23 needle.

The collected blood was placed in tubes with a coagulation activator (500 μl) and without it (50 μl). The tubes with the coagulation activator were incubated at room temperature for 20 minutes (in accordance with the protocol of the manufacturer of the tubes with the coagulation activator) and centrifuged for 20 minutes at 2000 g. The supernatant was transferred into a clean tube and stored at -80 °C until cytokine determination by ELISA assays of mouse TNFa and IL-6 DuoSet (R&D Systems, USA).

**Supplemental references**

1 Nishiyama, H. *et al.* Cloning and characterization of human CIRP (cold-inducible RNA-binding protein) cDNA and chromosomal assignment of the gene. *Gene* **204**, 115-120, doi:10.1016/s0378-1119(97)00530-1 (1997).

2 Liu, P. *et al.* Effects of Cold-inducible RNA-binding Protein (CIRP) on Liver Glycolysis during Acute Cold Exposure in C57BL/6 Mice. *Int J Mol Sci* **20**, doi:10.3390/ijms20061470 (2019).

3 Zhong, P., Peng, J., Yuan, M., Kong, B. & Huang, H. Cold-inducible RNA-binding protein (CIRP) in inflammatory diseases: Molecular insights of its associated signalling pathways. *Scand J Immunol* **93**, e12949, doi:10.1111/sji.12949 (2021).

4 Liu, M. *et al.* A novel target to reduce microglial inflammation and neuronal damage after deep hypothermic circulatory arrest. *J Thorac Cardiovasc Surg* **159**, 2431-2444 e2437, doi:10.1016/j.jtcvs.2019.06.115 (2020).

5 Liao, Y., Tong, L., Tang, L. & Wu, S. The role of cold-inducible RNA binding protein in cell stress response. *Int J Cancer* **141**, 2164-2173, doi:10.1002/ijc.30833 (2017).

6 De Leeuw, F. *et al.* The cold-inducible RNA-binding protein migrates from the nucleus to cytoplasmic stress granules by a methylation-dependent mechanism and acts as a translational repressor. *Exp Cell Res* **313**, 0014-4827, doi:10.1016/j.yexcr.2007.09.017 %M 17967451 (2007).

7 Qiang, X. *et al.* Cold-inducible RNA-binding protein (CIRP) triggers inflammatory responses in hemorrhagic shock and sepsis. *Nat Med* **19**, 1489-1495, doi:10.1038/nm.3368 (2013).

8 Zhou, Y. *et al.* The Cold-Inducible RNA-Binding Protein (CIRP) Level in Peripheral Blood Predicts Sepsis Outcome. *PLoS One* **10**, e0137721, doi:10.1371/journal.pone.0137721 (2015).

9 Yoo, I. S. *et al.* Serum and synovial fluid concentrations of cold-inducible RNA-binding protein in patients with rheumatoid arthritis. *Int J Rheum Dis* **21**, 148-154, doi:10.1111/1756-185X.12892 (2018).

10 Yang, W. L. *et al.* Cold-inducible RNA-binding protein causes endothelial dysfunction via activation of Nlrp3 inflammasome. *Sci Rep* **6**, 26571, doi:10.1038/srep26571 (2016).

11 Gong, J. D., Qi, X. F., Zhang, Y. & Li, H. L. Increased admission serum cold-inducible RNA-binding protein concentration is associated with prognosis of severe acute pancreatitis. *Clinica Chimica Acta* **471**, 135-142, doi:10.1016/j.cca.2017.06.002 (2017).

12 Linders, J. *et al.* Extracellular cold-inducible RNA-binding protein regulates neutrophil extracellular trap formation and tissue damage in acute pancreatitis. *Lab Invest* **100**, 1618-1630, doi:10.1038/s41374-020-0469-5 (2020).

13 Ode, Y. *et al.* Cold-inducible RNA-binding protein induces neutrophil extracellular traps in the lungs during sepsis. *Sci Rep* **9**, 6252, doi:10.1038/s41598-019-42762-1 (2019).

14 Zhou, M., Yang, W. L., Ji, Y., Qiang, X. & Wang, P. Cold-inducible RNA-binding protein mediates neuroinflammation in cerebral ischemia. *Biochim Biophys Acta* **1840**, 2253-2261, doi:10.1016/j.bbagen.2014.02.027 (2014).

15 Koch, R. E., Josefson, C. C. & Hill, G. E. Mitochondrial function, ornamentation, and immunocompetence. *Biol Rev Camb Philos Soc* **92**, 1459-1474, doi:10.1111/brv.12291 (2017).

16 Wi, S. M. *et al.* TAK1-ECSIT-TRAF6 complex plays a key role in the TLR4 signal to activate NF-kappaB. *J Biol Chem* **289**, 35205-35214, doi:10.1074/jbc.M114.597187 (2014).

17 Carneiro, F. R. G., Lepelley, A., Seeley, J. J., Hayden, M. S. & Ghosh, S. An essential role for ECSIT in mtochondrial complex Iassembly and mitophagy in macrophages. *Cell Rep* **22**, 2654-2666, doi:10.1016/j.celrep.2018.02.051 (2018).

18 Kopp, E. *et al.* ECSIT is an evolutionarily conserved intermediate in the Toll/IL-1 signal transduction pathway. *Genes Dev* **13**, 2059-2071, doi:10.1101/gad.13.16.2059 (1999).

19 Perkins, N. D. Integrating cell-signalling pathways with NF-kappaB and IKK function. *Nat Rev Mol Cell Biol* **8**, 49-62, doi:10.1038/nrm2083 (2007).

20 Horng, T., Barton, G. M. & Medzhitov, R. TIRAP: an adapter molecule in the Toll signaling pathway. *Nat Immunol* **2**, 835-841, doi:10.1038/ni0901-835 (2001).

21 Verstrepen, L. *et al.* TLR-4, IL-1R and TNF-R signaling to NF-kappaB: variations on a common theme. *Cell Mol Life Sci* **65**, 2964-2978, doi:10.1007/s00018-008-8064-8 (2008).

22 Tsung, A. *et al.* The nuclear factor HMGB1 mediates hepatic injury after murine liver ischemia-reperfusion. *J Exp Med* **201**, 1135-1143, doi:10.1084/jem.20042614 (2005).

23 Wu, H. *et al.* HMGB1 contributes to kidney ischemia reperfusion injury. *J Am Soc Nephrol* **21**, 1878-1890, doi:10.1681/ASN.2009101048 (2010).

24 Xue, J. *et al.* HMGB1 as a therapeutic target in disease. *J Cell Physiol* **236**, 3406-3419, doi:10.1002/jcp.30125 (2021).

25 Bertheloot, D. & Latz, E. HMGB1, IL-1alpha, IL-33 and S100 proteins: dual-function alarmins. *Cell Mol Immunol* **14**, 43-64, doi:10.1038/cmi.2016.34 (2017).

26 van Golen, R. F. *et al.* The damage-associated molecular pattern HMGB1 is released early after clinical hepatic ischemia/reperfusion. *Biochim Biophys Acta Mol Basis Dis* **1865**, 1879-1260X, doi:10.1016/j.bbadis.2019.01.014 %M 30658161 (2019).

27 Mangan, M. S. J. *et al.* Targeting the NLRP3 inflammasome in inflammatory diseases. *Nat Rev Drug Discov* **17**, 588-606, doi:10.1038/nrd.2018.97 (2018).

28 Gordon, A. C. *et al.* Interleukin-6 receptor antagonists in critically Ill patients with Covid-19. *N Engl J Med* **384**, 1491-1502, doi:10.1056/NEJMoa2100433 (2021).

29 Yang, H., Wang, H. & Andersson, U. Targeting inflammation driven by HMGB1. *Front Immunol* **11**, 484, doi:10.3389/fimmu.2020.00484 (2020).

30 Zhang, F., Brenner, M., Yang, W. L. & Wang, P. A cold-inducible RNA-binding protein (CIRP)-derived peptide attenuates inflammation and organ injury in septic mice. *Sci Rep* **8**, 3052, doi:10.1038/s41598-017-13139-z (2018).

31 Skulachev, V. P. Aging is a specific biological function rather than the result of a disorder in complex living systems: biochemical evidence in support of Weismann's hypothesis. *Biochemistry (Mosc)* **62**, 1191-1195 (1997).

32 Skulachev, V. P. Phenoptosis: programmed death of an organism. *Biochemistry (Mosc)* **64**, 1418-1426 (1999).

33 Skulachev, V. P. *et al.* Perspectives of Homo sapiens lifespan extension: focus on external or internal resources. *Aging (Albany NY)* **12**, 5566-5584, doi:10.18632/aging.102981 (2020).

34 Janeway, C. A. Approaching the asymptote? Evolution and revolution in immunology. *Cold Spring Harb Symp Quant Biol* **54 Pt 1**, 1-13, doi:10.1101/sqb.1989.054.01.003 (1989).

35 Barton, G. M. & Medzhitov, R. Toll-like receptors and their ligands. *Curr Top Microbiol Immunol* **270**, 81-92, doi:10.1007/978-3-642-59430-4_5 (2002).

36 Gong, T., Liu, L., Jiang, W. & Zhou, R. DAMP-sensing receptors in sterile inflammation and inflammatory diseases. *Nat Rev Immunol* **20**, 95-112, doi:10.1038/s41577-019-0215-7 (2020).

37 Denning, N. L., Aziz, M., Gurien, S. D. & Wang, P. DAMPs and NETs in Sepsis. *Front Immunol* **10**, 2536, doi:10.3389/fimmu.2019.02536 (2019).

38 Relja, B. & Land, W. G. Damage-associated molecular patterns in trauma. *Eur J Trauma Emerg Surg* **46**, 751-775, doi:10.1007/s00068-019-01235-w (2020).

39 Koonin, E. V. & Zhang, F. Coupling immunity and programmed cell suicide in prokaryotes: Life-or-death choices. *Bioessays* **39**, 1-9, doi:10.1002/bies.201600186 (2017).

40 Débarre, F., Lion, S., van Baalen, M. & Gandon, S. Evolution of host life-history traits in a spatially structured host-parasite system. *Am Nat* **179**, 52-63, doi:10.1086/663199 (2012).

41 Refardt, D. & Kümmerli, R. Defying bacteriophages: Contrasting altruistic with individual-based resistance mechanisms in Escherichia coli. *Commun Integr Biol* **6**, e25159, doi:10.4161/cib.25159 (2013).

42 Ferro, J. M. & Oliveira, S. Neurologic manifestations of gastrointestinal and liver diseases. *Curr Neurol Neurosci Rep* **14**, 487, doi:10.1007/s11910-014-0487-z (2014).

43 Arumugam, T. V. *et al.* Toll-like receptors in ischemia-reperfusion injury. *Shock* **32**, 4-16, doi:10.1097/SHK.0b013e318193e333 (2009).

44 Helms, J. *et al.* Neurologic Features in Severe SARS-CoV-2 Infection. *N Engl J Med* **382**, 2268-2270, doi:10.1056/NEJMc2008597 (2020).

45 Mehta, M. M., Weinberg, S. E. & Chandel, N. S. Mitochondrial control of immunity: beyond ATP. *Nat Rev Immunol* **17**, 608-620, doi:10.1038/nri.2017.66 (2017).

46 Mills, E. L., Kelly, B. & O'Neill, L. A. J. Mitochondria are the powerhouses of immunity. *Nat Immunol* **18**, 488-498, doi:10.1038/ni.3704 (2017).

47 Pinegin, B., Vorobjeva, N., Pashenkov, M. & Chernyak, B. The role of mitochondrial ROS in antibacterial immunity. *J Cell Physiol* **233**, 3745-3754, doi:10.1002/jcp.26117 (2018).

48 Vorobjeva, N. *et al.* Mitochondrial permeability transition pore is involved in oxidative burst and NETosis of human neutrophils. *Biochim Biophys Acta Mol Basis Dis* **1866**, 165664, doi:10.1016/j.bbadis.2020.165664 (2020).

49 Zhou, R., Yazdi, A. S., Menu, P. & Tschopp, J. A role for mitochondria in NLRP3 inflammasome activation. *Nature* **469**, 221-225, doi:10.1038/nature09663 (2011).

50 Bakeeva, L. E. *et al.* Mitochondria-targeted plastoquinone derivatives as tools to interrupt execution of the aging program. 2. Treatment of some ROS- and age-related diseases (heart arrhythmia, heart infarctions, kidney ischemia, and stroke). *Biochemistry (Mosc)* **73**, 1288-1299, doi:10.1134/s000629790812002x (2008).

51 Plotnikov, E. Y. *et al.* Protective effect of mitochondria-targeted antioxidants in an acute bacterial infection. *Proc Natl Acad Sci U S A* **110**, E3100-3108, doi:10.1073/pnas.1307096110 (2013).

52 Plotnikov, E. Y. *et al.* Mitochondrial damage and mitochondria-targeted antioxidant Pprotection in LPS-induced acute kidney injury. *Antioxidants (Basel)* **8**, doi:10.3390/antiox8060176 (2019).

53 Zakharova, V. V. *et al.* Low concentration of uncouplers of oxidative phosphorylation decreases the TNF-induced endothelial permeability and lethality in mice. *Biochim Biophys Acta Mol Basis Dis* **1863**, 968-977, doi:10.1016/j.bbadis.2017.01.024 (2017).

54 Skulachev, M. V. *et al.* Mitochondrial-targeted plastoquinone derivatives. Effect on senescence and acute age-related pathologies. *Curr Drug Targets* **12**, 800-826, doi:10.2174/138945011795528859 (2011).

55 Remolina, S. C., Chang, P. L., Leips, J., Nuzhdin, S. V. & Hughes, K. A. Genomic basis of aging and life-history evolution in Drosophila melanogaster. *Evolution* **66**, 3390-3403, doi:10.1111/j.1558-5646.2012.01710.x (2012).

56 Carnes, M. U. *et al.* The Genomic Basis of Postponed Senescence in Drosophila melanogaster. *PLoS One* **10**, e0138569, doi:10.1371/journal.pone.0138569 (2015).

57 Fabian, D. K. *et al.* Evolution of longevity improves immunity in. *Evol Lett* **2**, 567-579, doi:10.1002/evl3.89 (2018).

58 Kounatidis, I. *et al.* NF-κB Immunity in the Brain Determines Fly Lifespan in Healthy Aging and Age-Related Neurodegeneration. *Cell Rep* **19**, 836-848, doi:10.1016/j.celrep.2017.04.007 (2017).

59 Lin, Y. R., Parikh, H. & Park, Y. Stress resistance and lifespan enhanced by downregulation of antimicrobial peptide genes in the Imd pathway. *Aging (Albany NY)* **10**, 622-631, doi:10.18632/aging.101417 (2018).

60 Gorbunova, V., Seluanov, A. & Kennedy, B. K. The World Goes Bats: Living Longer and Tolerating Viruses. *Cell Metab* **32**, 31-43, doi:10.1016/j.cmet.2020.06.013 (2020).

61 Irving, A. T., Ahn, M., Goh, G., Anderson, D. E. & Wang, L. F. Lessons from the host defences of bats, a unique viral reservoir. *Nature* **589**, 363-370, doi:10.1038/s41586-020-03128-0 (2021).

62 Banerjee, A. *et al.* Novel Insights Into Immune Systems of Bats. *Front Immunol* **11**, 26, doi:10.3389/fimmu.2020.00026 (2020).

63 Goh, G. *et al.* Complementary regulation of caspase-1 and IL-1beta reveals additional mechanisms of dampened inflammation in bats. *Proc Natl Acad Sci U S A* **117**, 28939-28949, doi:10.1073/pnas.2003352117 (2020).

64 Salvioli, S. *et al.* Inflamm-aging, cytokines and aging: state of the art, new hypotheses on the role of mitochondria and new perspectives from systems biology. *Curr Pharm Des* **12**, 3161-3171, doi:10.2174/138161206777947470 (2006).

65 Millman, A. *et al.* Bacterial retrons function In anti-phage defense. *Cell* **183**, 1551-1561 e1512, doi:10.1016/j.cell.2020.09.065 (2020).

66 Rho, S. S., Ando, K. & Fukuhara, S. Dynamic regulation of vascular permeability by vascular endothelial cadherin-mediated endothelial cell-cell junctions. *J Nippon Med Sch* **84**, 148-159, doi:10.1272/jnms.84.148 (2017).

67 Uchimido, R., Schmidt, E. P. & Shapiro, N. I. The glycocalyx: a novel diagnostic and therapeutic target in sepsis. *Crit Care* **23**, 16, doi:10.1186/s13054-018-2292-6 (2019).

68 Ince, C. *et al.* The endothelium in sepsis. *Shock* **45**, 259-270, doi:10.1097/SHK.0000000000000473 (2016).

69 Andersson, U., Ottestad, W. & Tracey, K. J. Extracellular HMGB1: a therapeutic target in severe pulmonary inflammation including COVID-19? *Mol Med* **26**, 42, doi:10.1186/s10020-020-00172-4 (2020).

70 Chen, R. *et al.* HMGB1 as a potential biomarker and therapeutic target for severe COVID-19. *Heliyon* **6**, e05672, doi:10.1016/j.heliyon.2020.e05672 (2020).

71 Chen, L. *et al.* Elevated serum levels of S100A8/A9 and HMGB1 at hospital admission are correlated with inferior clinical outcomes in COVID-19 patients. *Cell Mol Immunol* **17**, 992-994, doi:10.1038/s41423-020-0492-x (2020).

72 Karkischenko, V. N. *et al.* Inhaled [D-Ala(2)]-Dynorphin 1-6 Prevents Hyperacetylation and Release of High Mobility Group Box 1 in a Mouse Model of Acute Lung Injury. *J Immunol Res* **2021**, 4414544, doi:10.1155/2021/4414544 (2021).

73 Hogeboom, G. H. Fractionation of cell components of animal tissues. *Methods Enzymol*, 16–19 (1955).

74 Vyssokikh, M. Y. *et al.* Mild depolarization of the inner mitochondrial membrane is a crucial component of an anti-aging program. *Proc Natl Acad Sci U S A* **117**, 6491-6501, doi:10.1073/pnas.1916414117 (2020).

75 Figueira, T. R., Melo, D. R., Vercesi, A. E. & Castilho, R. F. Safranine as a fluorescent probe for the evaluation of mitochondrial membrane potential in isolated organelles and permeabilized cells. *Methods Mol Biol* **810**, 103-117, doi:10.1007/978-1-61779-382-0_7 (2012).

76 Antonenko, Y. N. *et al.* Mitochondria-targeted plastoquinone derivatives as tools to interrupt execution of the aging program. 1. Cationic plastoquinone derivatives: synthesis and in vitro studies. *Biochemistry (Mosc)* **73**, 1273-1287, doi:10.1134/s0006297908120018 (2008).

77 Mei, J. *et al.* Body temperature measurement in mice during acute illness: implantable temperature transponder versus surface infrared thermometry. *Sci Rep-Uk* **8**, doi:10.1038/s41598-018-22020-6 (2018).

**Supplemental figures**


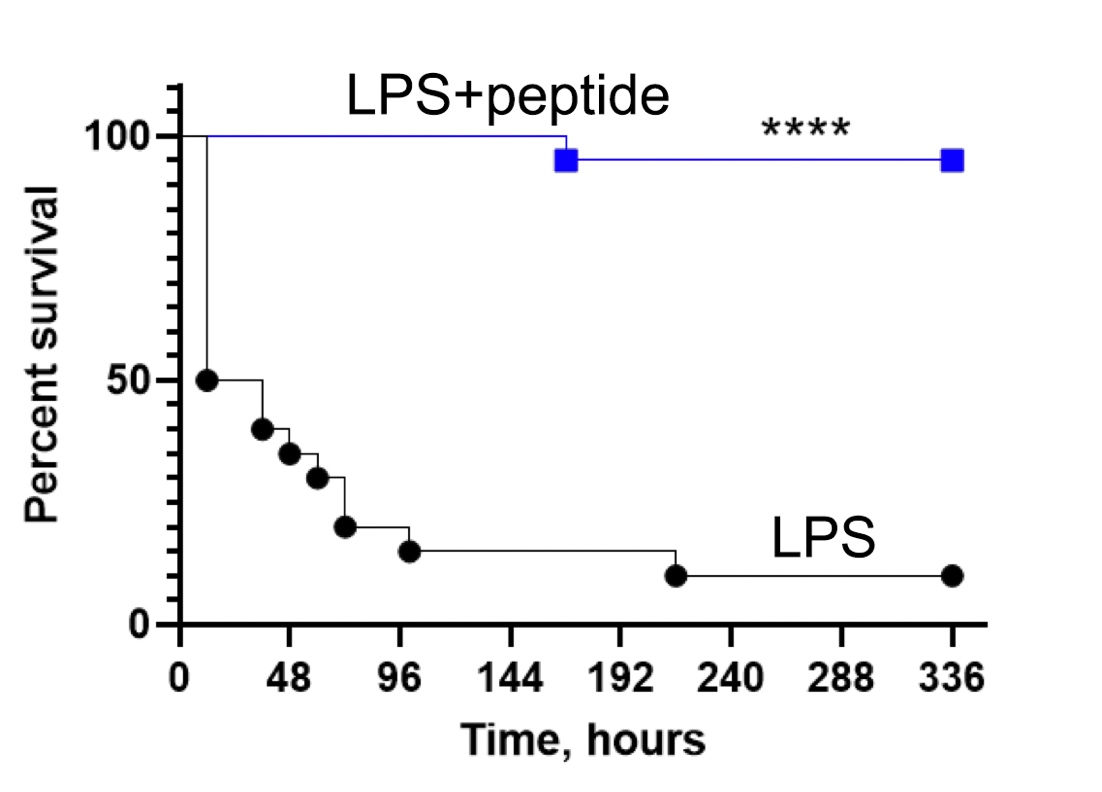


Fig. S1. A peptide called “leytragin” prevents killing mice by LPS (From Karkischenko et al.**^42^**).

**Table S1**. Data of Fig. 4C. The weights of mice (g) injected with mitochondria (with or without SkQ1). Deceased mice are shown in black boxes.

|  | **Days**    **Mouse N** | **0** | **1** | **2** | **3** | **4** | **5** | **6** | **7** | **8** | **9** | **10** | **11** | **12** | **13** | **14** | **15** | **16** |
| --- | --- | --- | --- | --- | --- | --- | --- | --- | --- | --- | --- | --- | --- | --- | --- | --- | --- | --- |
| Without SkQ1 | ***1*** | 30 | 31 | 26 | 25 | 27 | 28 | 28 | 29 | 29 | 30 | 31 | 32 | 30 | 32 | 33 | 33 | 35 |
|  | ***2*** | 33 | 33 | 29 |  |  |  |  |  |  |  |  |  |  |  |  |  |  |
|  | ***3*** | 29 | 28 | 24 | 22 | 22 | 24 | 25 | 26 | 27 | 27 | 27 | 28 | 28 | 28 | 28 | 28 | 30 |
|  | ***4*** | 27 | 26 | 24 | 25 | 26 | 26 | 26 | 28 | 28 | 28 | 29 | 30 | 30 | 31 | 32 | 33 | 32 |
|  | ***5*** | 27 | 26 | 22 | 20 | 18 | 21 | 21 | 22 | 23 | 24 | 24 | 26 | 26 | 26 | 26 | 27 | 29 |
|  | ***6*** | 31 |  |  |  |  |  |  |  |  |  |  |  |  |  |  |  |  |
|  | ***7*** | 24 |  |  |  |  |  |  |  |  |  |  |  |  |  |  |  |  |
|  | ***8*** | 32 | 32 | 27 | 24 | 24 | 26 | 27 | 27 | 28 | 29 | 30 | 30 | 31 | 31 | 32 | 33 | 34 |
|  | ***9*** | 29 |  |  |  |  |  |  |  |  |  |  |  |  |  |  |  |  |
|  | ***10*** | 26 | 24 | 21 | 20 | 21 | 24 | 25 | 25 | 25 | 26 | 26 | 26 | 26 | 26 | 27 | 28 | 30 |
| **Weight, mean** | | **28.8** | **28.6** | **24.7** | **22.7** | **23.0** | **24.8** | **25.3** | **26.2** | **26.7** | **27.3** | **27.8** | **28.7** | **28.5** | **29.0** | **29.7** | **30.3** | **31.7** |
| SkQ1 | ***1*** | 26 | 25 | 26 | 26 | 26 | 25 | 25 | 26 | 26 | 26 | 26 | 26 | 26 | 27 | 27 | 27 | 28 |
|  | ***2*** | 30 | 29 | 31 | 31 | 30 | 31 | 31 | 31 | 31 | 32 | 32 | 32 | 32 | 33 | 33 | 33 | 35 |
|  | ***3*** | 29 | 29 | 30 | 31 | 31 | 31 | 32 | 31 | 31 | 32 | 33 | 33 | 32 | 34 | 34 | 33 | 37 |
|  | ***4*** | 30 | 30 | 31 | 32 | 31 | 30 | 30 | 30 | 30 | 32 | 32 | 33 | 32 | 33 | 33 | 34 | 35 |
|  | ***5*** | 27 | 24 | 24 | 23 | 22 |  |  |  |  |  |  |  |  |  |  |  |  |
|  | ***6*** | 29 | 29 | 30 | 31 | 30 | 29 | 30 | 30 | 29 | 31 | 31 | 32 | 31 | 32 | 32 | 32 | 34 |
|  | ***7*** | 29 | 28 | 28 | 29 | 29 | 29 | 29 | 29 | 29 | 31 | 31 | 32 | 31 | 32 | 33 | 33 | 35 |
|  | ***8*** | 29 | 29 | 29 | 29 | 28 | 29 | 28 | 29 | 29 | 29 | 30 | 30 | 29 | 30 | 31 | 31 | 33 |
|  | ***9*** | 31 | 31 | 31 | 32 | 33 | 32 | 33 | 33 | 33 | 34 | 34 | 34 | 35 | 36 | 35 | 35 | 36 |
|  | ***10*** | 29 | 27 | 28 | 28 | 28 | 28 | 29 | 29 | 28 | 29 | 29 | 30 | 30 | 31 | 30 | 31 | 33 |
| **Weight, mean** | | **28.9** | **28.1** | **28.8** | **29.2** | **28.8** | **29.3** | **29.7** | **29.8** | **29.6** | **30.7** | **30.9** | **31.3** | **30.9** | **32.0** | **32.0** | **32.1** | **34.0** |

**Table S2.** Data in Fig. 5B. Weights of animals (g) subjected to cold stress with or without SkQ1. Deceased mice are shown in black boxes.

|  | Days  Mouse N | | **1** | **2** | **3** | **4** | **5** | **6** | **7** | **8** | **9** | **10** | **11** | **12** | **13** | **14** | **15** | **16** | **17** | **18** | **19** | **20** |
| --- | --- | --- | --- | --- | --- | --- | --- | --- | --- | --- | --- | --- | --- | --- | --- | --- | --- | --- | --- | --- | --- | --- |
| Without SkQ1 | ***1*** | | 43 | 42 | 43 | 39 | 43 | 42 | 37 | 34 | 31 |  | |  | |  | |  | |  | |  |
|  | ***2*** | | 43 | 40 | 43 | 41 | 42 | 41 | 39 | 40 | 39 | 39 | 40 | 38 | 37 | 41 | 39 | 40 | 43 | 43 | 44 | 45 |
|  | ***3*** | | 42 | 39 | 42 | 42 | 43 | 40 | 41 | 41 | 40 | 42 | 42 | 40 | 38 | 39 | 39 | 38 | 43 |  |  |  |
|  | ***4*** | | 40 | 35 | 40 | 39 | 40 | 40 | 40 | 39 | 36 | 35 | 35 | 33 | 32 | 35 | 36 | 38 | 39 |  |  |  |
|  | ***5*** | | 44 | 42 | 44 | 43 | 43 | 41 | 43 | 42 | 40 | 39 | 37 | 38 | 39 | 41 | 39 | 40 | 42 | 43 | 44 | 45 |
|  | ***6*** | | 39 | 36 | 39 | 35 | 38 | 38 | 34 | 39 | 28 |  | |  | |  | |  | |  | |  |
|  | ***7*** | | 39 | 38 | 39 | 36 | 39 | 38 | 35 | 32 | 32 | 30 | 30 | 29 | 27 | 30 | 31 | 32 | 35 | 36 | 38 | 40 |
|  | ***8*** | | 47 | 44 | 47 | 46 | 47 | 45 | 44 | 44 | 42 | 39 | 38 | 38 | 39 | 39 | 40 | 42 | 45 | 46 | 48 | 50 |
|  | ***9*** | | 41 | 39 | 41 | 37 | 40 | 39 | 35 | 32 | 29 |  | |  | |  | |  | |  | |  |
|  | ***10*** | | 39 | 37 |  |  |  |  |  |  |  |  |  |  |  |  |  |  |  |  |  |  |
| **Weight, mean** | | | **41.7** | **39.2** | **42.0** | **39.8** | **41.7** | **40.4** | **38.7** | **38.1** | **35.2** | **37.3** | **37.0** | **36.0** | **35.3** | **37.5** | **37.3** | **38.3** | **41.2** | **42.0** | **43.5** | **45.0** |
| SkQ1 | | ***1*** | 42 | 39 | 42 | 40 | 40 | 39 | 40 | 39 | 39 | 40 | 37 | 39 | 40 | 40 | 41 | 41 | 42 | 42 | 42 | 43 |
|  |  | ***2*** | 36 | 34 | 36 | 36 | 37 | 37 | 37 | 37 | 38 | 39 | 38 | 38 | 38 | 39 | 37 | 36 | 36 | 38 | 37 | 38 |
|  |  | ***3*** | 39 | 39 | 39 | 39 | 41 | 41 | 40 | 42 | 41 | 41 | 39 | 40 | 42 | 41 | 42 | 42 | 43 | 42 | 41 | 40 |
|  |  | ***4*** | 42 | 40 | 42 | 40 | 44 | 44 | 44 | 44 | 44 | 44 | 41 | 43 | 44 | 44 | 43 | 44 | 44 | 44 | 44 | 45 |
|  |  | ***5*** | 39 | 37 | 39 | 39 | 40 | 40 | 40 | 39 | 40 | 40 | 39 | 41 | 42 | 41 | 42 | 40 | 42 | 44 | 41 | 43 |
|  |  | ***6*** | 43 | 42 | 43 | 44 | 44 | 44 | 46 | 45 | 44 | 44 | 43 | 42 | 44 | 44 | 45 | 41 | 45 | 48 | 44 | 47 |
|  |  | ***7*** | 41 | 39 | 41 | 41 | 41 | 42 | 42 | 42 | 42 | 40 | 39 | 40 | 40 | 40 | 40 | 41 | 41 | 43 | 43 | 43 |
|  |  | ***8*** | 36 | 35 | 36 | 36 | 39 | 39 | 39 | 40 | 40 | 40 | 38 | 39 | 39 | 40 | 40 | 41 | 41 | 41 | 41 | 41 |
|  |  | ***9*** | 42 | 40 | 42 | 40 | 42 | 42 | 41 | 41 | 41 | 42 | 41 | 35 | 37 | 39 | 39 | 38 | 41 | 42 | 43 | 43 |
| **Weight, mean** | | | **40.0** | **38.3** | **40.0** | **39.4** | **40.9** | **40.9** | **41.0** | **41.0** | **41.0** | **41.1** | **39.4** | **39.7** | **40.7** | **40.9** | **41.0** | **40.4** | **41.7** | **42.7** | **41.8** | **42.6** |

**Table S3.** Dynamics of body temperature (°С ) in mice without SkQ1 and with SkQ1. The time at the outer temperature –20 °С or at room temperature (highlighted in blue or in red, respectively). Data of Fig. 6C.

|  | **Mouse**  **N_**  **Min___** | **1** | **2** | **3** | **4** | **5** | **6** | **7** | **8** | **9** | **10** |
| --- | --- | --- | --- | --- | --- | --- | --- | --- | --- | --- | --- |
| Without SkQ1 | **0** | 37.9 | 38.1 | 37.6 | 39.2 | 38.3 | 37.6 | 38.8 | 39.1 | 38.3 | 37.8 |
|  | **10** | 37.6 | 37.7 | 38.2 | 37.4 | 38.1 | 38.7 | 39.2 | 38.6 | 38.7 | 38.1 |
|  | **20** | 37.1 | 36.8 | 37.2 | 36.4 | 37.2 | 36.1 | 36.9 | 36 | 37.2 | 37.4 |
|  | **30** | 35.3 | 34.8 | 35.1 | 36.1 | 34.9 | 35.2 | 34.8 | 33.9 | 36.5 | 34.3 |
|  | **40** | 31.2 | 32.4 | 33.1 | 32.9 | 31.3 | 34.2 | 30.4 | 32.5 | 33.1 | 30.9 |
|  | **50** | 26.8 | 25.7 | 28.4 | 30.1 | 27.6 | 29.3 | 24.4 | 28.2 | 27.8 | 25.1 |
|  | **60** | 23.6 | 22.9 | 21.6 | 23.8 | 22.7 | 23.3 | 21.9 | 24.1 | 23.2 | 22.8 |
|  | **70** | 20.4 | 19.8 | 20.3 | 19.4 | 21.8 | 20.6 | 20.7 | 21.2 | 20.5 | 21.3 |
|  | **80** | 19.4 | 18.7 | 18.2 | 20.2 | 22.3 | 20.2 | 22.8 | 19.3 | 18.9 | 20.4 |
|  | **90** | 18.7 | 19.1 | 19.8 | 18.9 | 27.4 | 18.4 | 28.5 | 19.8 | 19.2 | 18.2 |
|  | **100** | 19.6 | 18.4 | 19.4 | 19.7 | 34.3 | 19.6 | 32.9 | 19.1 | 19.4 | 18.8 |
|  | **110** | 20.2 | 19.5 | 18.7 | 19.1 | 37.8 | 20.1 | 34.6 | 18.6 | 18.8 | 18.6 |
|  | **120** | 18.3 | 19.1 | 19.5 | 18.8 | 37.9 | 18.4 | 38.3 | 19.4 | 19.2 | 19.5 |
| SkQ1 | **Mouse**  **N_**  **Min__** | **1** | **2** | **3** | **4** | **5** | **6** | **7** | **8** | **9** | **10** |
|  | **0** | 38.6 | 39.4 | 37.9 | 38.5 | 39.1 | 39.3 | 37.9 | 39 | 38.3 | 37.8 |
|  | **10** | 38.1 | 37.6 | 38.2 | 37.8 | 38.3 | 37.4 | 38.2 | 38.2 | 37.4 | 38.5 |
|  | **20** | 37.4 | 34.8 | 36.4 | 37.1 | 36.5 | 37.5 | 37.4 | 38.4 | 38.1 | 37.4 |
|  | **30** | 36.2 | 32.1 | 35.5 | 36.5 | 34.2 | 33.9 | 35.9 | 35.1 | 34.5 | 35.2 |
|  | **40** | 32.3 | 30.4 | 30.9 | 34.8 | 32.6 | 33.1 | 32.5 | 30.4 | 31.3 | 30.8 |
|  | **50** | 28.1 | 25.2 | 28.6 | 31.7 | 25.4 | 28.2 | 27.4 | 26.5 | 25.4 | 26.1 |
|  | **60** | 25.4 | 22.4 | 23.4 | 24.2 | 22.1 | 23.9 | 22.8 | 22.8 | 23.7 | 22.3 |
|  | **70** | 26.7 | 27.3 | 25.9 | 28.4 | 27.8 | 21.1 | 28.2 | 19.6 | 20.2 | 26.9 |
|  | **80** | 29.3 | 31.4 | 32.7 | 30.5 | 32.6 | 20.4 | 32.6 | 18.8 | 18.9 | 30.8 |
|  | **90** | 34.8 | 36.7 | 38.3 | 36.9 | 37.8 | 18.8 | 35.8 | 19.3 | 18.6 | 36.7 |
|  | **100** | 37.1 | 38.4 | 39.8 | 38.7 | 39.5 | 19.6 | 38.9 | 19.1 | 19.4 | 39.4 |
|  | **110** | 39.2 | 39.7 | 38.7 | 39.4 | 39.8 | 19.1 | 39.3 | 18.7 | 19.8 | 39.1 |
|  | **120** | 38.4 | 38.8 | 39.1 | 39.5 | 38.7 | 18.8 | 38.1 | 18.9 | 18.7 | 39.3 |

**Table S4.** Dynamics of changes in the TNF-α and IL-6 amounts in the blood of mice after injection of mitochondria into a tail vein. SkQ1 (1.5 μmol/kg of animal weight) was intraperitoneally injected for 5 days before the mitochondrial injection. Data are given for 3 hours after mitochondrial injection.

| ***TNF-α, pg/ml*** | **Without**  **SkQ1** | **SkQ1** |
| --- | --- | --- |
|  | 188 | 26 |
|  | 191 | 52 |
|  | 176 | 38 |
|  | 204 | 150 |
|  | 213 | 16 |
|  | 155 | 125 |
| **Mean±SD**  *p<0.01* | ***188±21*** | ***68±55*** |
| ***IL-6, pg/ml*** | **Without**  **SkQ1** | **SkQ1** |
|  | 1824 | 82 |
|  | 1797 | 364 |
|  | 1659 | 236 |
|  | 1575 | 1841 |
|  | 1853 | 124 |
|  | 1717 | 850 |
| **Mean±SD**  *p<0.05* | ***1737±107*** | ***583±676*** |

**Table S5.** Dynamics of changes in IL-6 concentration in the blood of mice after cooling.

| **Hours** | **Without SkQ1** | **SkQ1** |
| --- | --- | --- |
| **4** | 1118 | 4 |
|  | 1110 | 6 |
|  | 1347 | 4 |
|  | 1309 | 689 |
| **Mean±SD** | **1221*±125*** | **176*±342*** |
| **8^*^**  ***p<0.05***  ***for SkQ1*** | 1147 | 643 |
|  | 1191 | 1 |
|  | 1072 | Not detected |
|  | 1048 | Not detected |
|  | 1194 | Not detected |
|  | 1255 |  |
| **Mean±SD** | **1151*±79*** | **161*±321*** |
| **12^*^**  ***p<0.05***  ***for SkQ1*** | 1364 | 1 |
|  | 1344 | Not detected |
|  | 1206 | 4 |
|  | 1167 | 3 |
|  | 1091 | Not detected |
| **Mean±SD** | **1234*±117*** | **2*±2*** |
| **15** | 1477 | Not detected |
|  | 1352 | Not detected |
|  | 1599 | Not detected |
|  | 1515 | 978 |
|  | 1339 |  |
| **Mean±SD** | **1456*±111*** | **245*±489*** |

**Table S6.** Dynamics of changes in IL-6 concentration in the blood of mice after injection of C_12_TPP into a tail vein (34 µmol / kg of animal weight). SkQ1 (1.5 μmol/kg of animal weight) or a solution without SkQ1 was injected intraperitoneal daily for 5 days before the injection of C_12_TPP. Data are given for 1, 12 and 24 hours after C_12_TPP injection.

| **Hours** | **Without SkQ1** | **SkQ1** |
| --- | --- | --- |
| **0** | 18 | 19 |
|  | 20 | 17 |
|  | 20 | 20 |
|  | 19 | 17 |
|  | 20 | 17 |
|  | 20 | 18 |
| **Mean±SD** | **20*±0.8*** | **18*±1.3*** |
| **1** | 1099 | Not detected |
|  | 1 | Not detected |
|  | Not detected | 1116 |
|  | Not detected | Not detected |
|  | Not detected | Not detected |
|  | Not detected | Not detected |
| **Mean±SD** | **183±448.6** | **186±455.6** |
| **12^*^**  ***p<0.01*** | 1146 | 7 |
|  | 1395 | 19 |
|  | 1128 | 18 |
|  | 1011 | 18 |
|  | 940 | 18 |
|  | 1042 | 18 |
|  | 1303 | 1097 |
|  | 1266 | 1142 |
|  | 1148 | Not detected |
|  | 1137 | Not detected |
|  | 1062 | Not detected |
|  | 1002 | Not detected |
| **Mean±SD** | **1132±134.3** | **195±432.1** |
| **24** | 1146 | Not detected |
|  | 1193 | Not detected |
|  | 1317 | Not detected |
|  | 1264 | Not detected |
|  | Not detected | 164 |
|  | Not detected |  |
| **Mean±SD** | **820±637.9** | **33±73.3** |
